# A 6-month inhalation toxicology study in *Apoe*^*−/−*^ mice demonstrates substantially lower effects of e-vapor aerosol compared with cigarette smoke in the respiratory tract

**DOI:** 10.1007/s00204-021-03020-4

**Published:** 2021-05-07

**Authors:** Ee Tsin Wong, Justyna Szostak, Bjoern Titz, Tom Lee, Sin Kei Wong, Oksana Lavrynenko, Celine Merg, Maica Corciulo, Jovan Simicevic, Mehdi Auberson, Dariusz Peric, Remi Dulize, David Bornand, Guo Jie Loh, Kyeonghee Monica Lee, Jingjie Zhang, John H. Miller, Walter K. Schlage, Emmanuel Guedj, Thomas Schneider, Blaine Phillips, Patrice Leroy, Mohamed Amin Choukrallah, Nicolas Sierro, Ansgar Buettner, Yang Xiang, Arkadiusz Kuczaj, Nikolai V. Ivanov, Karsta Luettich, Patrick Vanscheeuwijck, Manuel C. Peitsch, Julia Hoeng

**Affiliations:** 1 PMI R&D, Philip Morris International Research Laboratories Pte. Ltd, Science Park II, Singapore, 117406 Singapore; 2 PMI R&D, Philip Morris Products S.A, Quai Jeanrenaud 5, 2000 Neuchâtel, Switzerland; 3 Altria Client Services LLC, 601 East Jackson Street, Richmond, VA 23219 USA; 4 Max-Baermann-Str. 21, 51429 Bergisch Gladbach, Germany; 5 Histovia GmbH, Schöne Aussicht 5, 51491 Overath, Germany

**Keywords:** Electronic cigarette, Smoking, Emphysema, Inflammation, COPD

## Abstract

Cigarette smoking is the major cause of chronic obstructive pulmonary disease. Considerable attention has been paid to the reduced harm potential of nicotine-containing inhalable products such as electronic cigarettes (e-cigarettes). We investigated the effects of mainstream cigarette smoke (CS) and e-vapor aerosols (containing nicotine and flavor) generated by a capillary aerosol generator on emphysematous changes, lung function, and molecular alterations in the respiratory system of female *Apoe*^−/−^ mice. Mice were exposed daily (3 h/day, 5 days/week) for 6 months to aerosols from three different e-vapor formulations—(1) carrier (propylene glycol and vegetable glycerol), (2) base (carrier and nicotine), or (3) test (base and flavor)—or to CS from 3R4F reference cigarettes. The CS and base/test aerosol concentrations were matched at 35 µg nicotine/L. CS exposure, but not e-vapor exposure, led to impairment of lung function (pressure–volume loop area, A and K parameters, quasi-static elastance and compliance) and caused marked lung inflammation and emphysematous changes, which were confirmed histopathologically and morphometrically. CS exposure caused lung transcriptome (activation of oxidative stress and inflammatory responses), lipidome, and proteome dysregulation and changes in DNA methylation; in contrast, these effects were substantially reduced in response to the e-vapor aerosol exposure. Compared with sham, aerosol exposure (carrier, base, and test) caused a slight impact on lung inflammation and epithelia irritation. Our results demonstrated that, in comparison with CS, e-vapor aerosols induced substantially lower biological and pathological changes in the respiratory tract associated with chronic inflammation and emphysema.

**Supplementary Information:**

The online version supplementary material available at 10.1007/s00204-021-03020-4.

## Introduction

Chronic obstructive pulmonary disease (COPD) is a major global health problem and is among the leading causes of morbidity and mortality (Lopez-Campos et al. 2016; Quaderi and Hurst 2018). COPD is defined as a “preventable and treatable disease that is characterized by persistent respiratory symptoms and airflow limitation that is due to airway and/or alveolar abnormalities usually caused by significant exposure to noxious particles or gases” (GOLD-COPD 2019). The airflow limitation is progressive and strongly associated with airway inflammation from macrophage, neutrophil, and T-cell infiltration in the lungs, leading to mucus hypersecretion, airway remodeling, emphysema, reduced lung function, and dyspnea (Barnes 2017; Butler et al. 2018; Demedts et al. 2006; Higham et al. 2019; Hogg 2004; Huang et al. 2017; Stratton et al. 2001; Tetley 2005; Wang et al. 2018). Cigarette smoking is the predominant cause of COPD in industrialized countries and accounts for more than 95% of cases (Barnes et al. 2003; Buist et al. 2008; Churg et al. 2008). Cigarette smoke (CS) exposure exacerbates and perpetuates inflammation, causing airway remodeling, airway obstruction, and emphysematous changes characteristic of COPD (Barnes et al. 2003; Churg et al. 2008; Ghorani et al. 2017; Leberl et al. 2013; Office of the Surgeon General U. S. 2010).

To minimize the adverse effects caused by cigarette smoking, alternative nicotine-delivery products are being developed for adult smokers. For example, electronic cigarettes (e-cigarettes or e-vapor products)—considered as one of potential RRPs—deliver nicotine in an aerosol without the combustion products that are responsible for most of damaging effects of CS (Farsalinos et al. 2015, 2013; Polosa and Caponnetto 2013a, 2013b).

While toxicological investigations of e-vapor aerosols are relatively recent, the currently available evidence indicates that the aerosols generated by e-cigarettes are less harmful than CS, and significantly reduced COPD-related changes (such as inflammation and lung function decline) are expected in smokers who completely switch from cigarettes to e-cigarettes (Farsalinos and Polosa 2014; Hajek 2014; Polosa et al. 2011, 2018). E-vapor formulations (e-liquids) are typically composed of carriers (propylene glycol [PG] and vegetable glycerol [VG]; ~  > 90%), nicotine (~ 5%), and flavor mixtures. In rats inhalation studies, PG, VG, PG/VG, and PG/VG/nicotine aerosols demonstrated significantly lower pulmonary toxicity than CS (Olfert et al. 2018; Phillips et al. 2017; Renne et al. 1992; Suber et al. 1989; Werley et al. 2011, 2016). However, even though the flavor compounds used in e-cigarette liquids are generally regarded as safe (GRAS) for use in food products, their toxicity when inhaled requires comprehensive characterization.

The objective of this study was to assess the impact of exposure to unflavored and flavored e-vapor aerosols generated using a capillary aerosol generator on the respiratory system and to comparatively evaluate COPD-related changes (pulmonary inflammation, emphysematous changes, lung function), as well as the underlying molecular changes relative to those observed after exposure to CS in *Apoe*^−/−^ mice.

## Materials and methods

### Study design

The animals were approximately 12–14 weeks old at the start of exposure and were randomly allocated to five exposure groups (Online Resource 1a): sham (exposed to fresh air); 3R4F reference CS (“3R4F”); an aerosol containing PG and VG (“carrier”); an aerosol containing PG, VG, and nicotine (“base”); and an aerosol containing PG, VG, nicotine, and flavoring (“test”). To the nicotine-containing (4% [w/w]) base and test formulations, mixtures of acids (1% [w/w]) were added to attain a pH of ~ 8.0 (Online Resource 1a). The animals were exposed to 3R4F CS or e-vapors in whole-body exposure chambers for a total of 3 h per day, 5 days per week, with a 30-min fresh-air break after the first hour of exposure and a 60-min break after the second hour (Online Resource 1b). The maximum exposure duration was 6 months, and dissections were scheduled after 3 and 6 months.

The base and test exposure atmospheres were configured to deliver the same concentration of nicotine as the 3R4F CS (nicotine concentration of 35 µg/L, corresponding to 560 µg/L total particulate matter [TPM]. The carrier group exposure was configured to deliver the same concentration of TPM as the base and the test groups (Online Resource 1c, Online Resource 2).

### Test atmosphere generation

3R4F reference cigarettes were purchased from the University of Kentucky (https://ctrp.uky.edu/home). Mainstream smoke from 3R4F cigarettes was generated on 30-port rotary smoking machines (SM2000, PMI R&D, Neuchâtel, Switzerland) in accordance with the Health Canada Intense Smoking Protocol (Health-Canada 1999). The 3R4F puff count was 10–11 per stick. The smoking machine and chamber layout for sham and 3R4F exposure conditions are shown in Online Resource 3. Mice in the sham group were exposed to fresh air through a smoking machine without cigarettes.

Nicotine represents 4% of the total concentration in the base and test formulations. A blended flavor mix represents 0.12% of the total concentration in the test formulation (Online Resource 4 and Online Resource 5). The carrier, base, and test laboratory aerosols were generated using a capillary aerosol generator (CAG; Online Resource 6) set to 250–275℃, a typical temperature range of the heated coil during puffing of an e-cigarette (Geiss et al. 2016). Additional information can be found in Online Resource 2.

### Animals and inhalation exposure

All procedures involving animals were performed in a facility accredited by the Association for Assessment and Accreditation of Laboratory Animal Care (AAALAC) and licensed by the Singapore National Parks/Animal and Veterinary Service (AVS), with approval from an Institutional Animal Care and Use Committee and in compliance with the National Advisory Committee for Laboratory Animal Research Guidelines on the Care and Use of Animals for Scientific Purposes (NACLAR 2004). Female *Apoe*^−/−^ mice (B6.129P2-*ApoE*^*tm1/Unc*^ N11) bred under specific pathogen-free conditions were obtained from Taconic Biosciences (Rensselaer, NY, USA).

The mice were whole-body exposed to diluted mainstream smoke from 3R4F cigarettes (target concentration: 560 µg TPM/L, equivalent to 35.9 µg nicotine/L). This nicotine concentration was matched in the base and test aerosols, and this base and test TPM concentration was matched in the carrier and test aerosols (Online Resource 1a). Intermittent exposures to fresh filtered air for 30 and 60 min after the first and second hours of exposure, respectively, were provided to avoid buildup of excessive carboxyhemoglobin in the 3R4F CS-exposed mice (Online Resource 1b).

### Analysis of carbonyls and tobacco-specific nitrosamines in the test atmospheres

Carbonyl compound concentrations were assessed by ALCS (Richmond, USA). Formaldehyde, acetaldehyde, crotonaldehyde, propionaldehyde, and acrolein concentrations in the test atmospheres were assessed by liquid chromatography–mass spectrometry (LC–MS) of the corresponding 2,4-dinitrophenylhydrazine (DNPH) derivatives after trapping in DNPH solution. Three test atmosphere samples were collected monthly to assess the mean levels of carbonyls in the exposure chambers.

The concentrations of tobacco-specific nitrosamines (*N*-nitrosonornicotine [NNN] and 4-(methylnitrosamino)1-(3-pyridyl)-1-butanone [NNK]) in the nicotine stock and inhalation formulations were assessed (once during study) by LC–tandem MS (LC–MS/MS) by ABF GmbH (Planegg, Germany).

Carbon monoxide was analyzed continuously by nondispersive infrared photometry of the gas/vapor phase using a carbon monoxide meter (Ultramat 6E, Siemens N.V., Brussels, Belgium) placed in the pipe leading to the exposure chamber. Guaiacol concentration was determined by gas chromatography–mass spectrometry (GC–MS; TÜV SÜD PSB Pte. Ltd., Singapore).

### Analysis of nicotine, cotinine, and PG as biomarkers of exposure in blood and plasma

Blood samples from 8 mice per group per time point (months 1 and 4 of the study) were collected within 15 min following a 3-h exposure. For plasma preparation, blood was placed on ice after collection and processed. Aliquoted plasma was transferred to storage at ≤ –70℃. Plasma PG, nicotine, and cotinine levels were measured using LC–MS/MS (Meger et al. 2002; Scherer et al. 2007) at ABF GmbH.

### Analysis of nicotine and total nicotine metabolites in urine

After 1 month of exposure, urine was collected during exposure by placing individual mice in exposure cages with a raised bottom grid and in the 18-h period following the exposure in a urine metabolic cage. Urine collected during exposure, urine from the 18-h overnight collection, and water from rinsing of the cage (approximately 100 µL) were pooled per animal, aliquoted, and stored at ≤ –70℃. Analysis of nicotine metabolites (trans-3′-hydroxycotinine, norcotinine, cotinine, nicotine-*N′*-oxide, and nornicotine) in urine was performed by LC–MS/MS after 1,3-diethyl-2-thiobarbituric acid derivatization at ABF GmbH.

The same samples were analyzed for other urinary non-nicotine biomarkers such as 3-hydroxypropyl mercapturic acid (HPMA), *S*-phenylmercapturic acid (SPMA), 2-cyanoethylmercapturic acid (CEMA), the two isoforms of monohydroxybutenylmercapturic acid (1-MHBMA and 2-MHBMA) by ABF GmbH.

### Lung function and lung volume measurements

Lung function measurements were performed in anesthetized, tracheotomized, and cannulated mice (from the Histopathology group; Online Resource 2c) using the flexiVent™ rodent ventilator system for measurement of respiratory mechanics (SCIREQ, Montreal, QC, Canada) as described previously (Phillips et al. 2015). Lung volume was determined by the fluid displacement method in the lungs of mice scheduled for histopathological analysis (*n* = 12) (Online Resource 1c) (Scherle 1970).

### BALF collection

BALF was collected from 10 mice per group (Online Resource 1c). The numbers and types of free lung cells in the BALF were determined, and the proteolytic activity and concentrations of inflammatory mediators were measured. Details of the methods were described previously (Boue et al. 2013).

### Histopathological analysis and morphometry

Histopathological analysis and morphometry were performed in mice from the Histopathology group (c). The lungs were fixed by instillation with 4% (w/v) formaldehyde (aq.) (pH 7.4) at 20 cm H_2_O fixed pressure and processed as described previously (Boue et al. 2013). Histopathological evaluation of the left lung (serial sections), nose, larynx, and trachea was performed in a blinded fashion by a board-certified veterinary pathologist (Histovia GmbH, Overath, Germany). Findings were recorded as incidences and/or a semi-quantitative severity grading in five steps. The histopathological score definitions were: (0) normal, (1) minimal; (2) mild; (3) moderate; (4) marked; and (5) severe.

### Respiratory tract tissue processing

Tissues for molecular analysis of the lungs, respiratory nasal epithelium, and trachea were collected at months 3 and 6. Tissue dedicated to molecular analysis (*n* = 10 per time point and group) were collected as described previously (Phillips et al. 2016).

### Lung, respiratory nasal epithelium, and trachea transcriptomics analyses

Samples were randomized to ensure balanced assignment of the experimental groups across the RNA extraction batches and Affymetrix hybridization Fluidics Stations (Santa Clara, CA, USA). Total RNA was isolated from tissues using a miRNeasy Mini Kit (Qiagen, Hilden, Germany). RNA concentrations were assessed using a NanoDrop ND-1000 spectrophotometer (Thermo Fisher Scientific), and an Agilent 2100 Bioanalyzer was used to assess RNA quality (Agilent Technologies, Santa Clara, CA, USA). The data were deposited in the ArrayExpress database (https://www.ebi.ac.uk/arrayexpress/) under accession number E-MTAB-8578.

Transcriptomic data from the lung, respiratory nasal epithelium, and tracheal tissues were also analyzed in the context of hierarchically structured network models describing the molecular mechanisms underlying essential biological processes in non-diseased lungs (Boue et al. 2015). By leveraging the cause-and-effect network models together with network perturbation amplitude (NPA) algorithms, the gene expression fold changes were translated into differential values for each network node, which were, in turn, summarized into a quantitative NPA measure, and NPA values from all applied network models were aggregated into a biological impact factor (BIF) score (Hoeng et al. 2012; Martin et al. 2014). Application of BIF scores in inhalation studies has been described in detail elsewhere (Kogel et al. 2014; Phillips et al. 2015). The relative BIF is the BIF value normalized (as a percentage) to the maximum BIF of the study.

### Lung lipidomics analysis

Lipidomics analyses were performed (*n* = 10; Online Resource 1c) using a high-resolution MS/MS shotgun lipidomics protocol as described in. The Benjamini–Hochberg FDR method was used to correct for multiple testing effects. Lipids with an adjusted *p* value < 0.05 were considered differentially abundant.

### Lung proteomics analysis

Proteome alterations in the lungs (*n* = 10) were assessed by isobaric-tag-based quantification using the iTRAQ® approach as described previously (Titz et al. 2015) (Details in Online Resource 2).

For the statistical analysis, a linear model was fitted for each exposure condition and the respective sham group. *p* values were calculated from moderated *t*-statistics with the empirical Bayes approach, and proteins with a Benjamini–Hochberg FDR-adjusted *p* value < 0.05 were considered differentially expressed (Gentleman et al. 2004).

### Lung whole-genome methylation analysis

DNA isolation and quantification were performed as described previously (Phillips et al. 2019). Whole-genome bisulfite sequencing libraries were prepared using an Ovation® Ultralow Methyl-Seq Library Systems kit (#0541–32; Tecan, Männedorf, Switzerland) and sequenced using the Illumina HiSeq4000 (Illumina Inc., San Diego, CA, USA).

### Statistical analysis for apical endpoints

A statistical approach leveraging two-sample tests was implemented, which enabled us to perform consistent analysis across all endpoints (Phillips et al. 2019, 2017, 2016). Data are expressed as mean ± standard error of the mean. Pairwise comparisons between groups were performed, and unadjusted *p* values are reported. The results were considered significantly different for a specific comparison if *p* < 0.05.

Additional information about materials and methods is available in Online Resource 2.

### Data availability

Datasets and additional data visualizations can be accessed at https://doi.org/org/10.26126/intervals.8lafdu.1.

## Results

### E-vapors contain lower levels of carbonyls and harmful and potentially harmful constituents (HPHCs) than 3R4F CS, leading to reduced exposure and uptake to HPHCs

Carbonyl (formaldehyde, acetaldehyde, propionaldehyde, crotonaldehyde, and acrolein) levels were consistently higher in the 3R4F CS atmosphere than in the sham atmosphere (Table 1). Compared with 3R4F CS, aerosols from the carrier, base, and test formulations contained much lower levels of carbonyl compounds (Table 1). The concentrations of formaldehyde, acetaldehyde, and propionaldehyde in the base and test aerosols were over 95% lower than those in 3R4F CS at equal nicotine concentrations. Acrolein and crotonaldehyde levels were at the background levels (similar to sham) in the e-vapor aerosol groups, in contrast to concentrations of 3.98 and 2.84 µg/L, respectively, in 3R4F CS.

**Table 1:** Test atmosphere characterization and composition of e-vapor aerosols (carrier, base, and test) and 3R4F CS

	Endpoints	Unit	Sham	3R4F	CARRIER (PG/VG)	BASE (PG/VG/N)	TEST (PG/VG/N/F)
Aerosol constituants	Formaldehyde	µg/L	0.017	0.665	0.028	0.036	0.028
			*(*± *0.01)*	*(*± *0.08)*	*(*± *0.01)*	*(*± *0.03)*	*(*± *0.01)*
	Acetaldehyde	µg/L	0.009	30.686	0.012	0.022	0.02
			*(*± *0.00)*	*(*± *1.90)*	*(*± *0.00)*	*(*± *0.01)*	*(*± *0.01)*
	Propionaldehyde	µg/L	0.001	3.471	0.007	0.007	0.007
			*(*± *0.00)*	*(*± *0.62)*	*(*± *0.00)*	*(*± *0.00)*	*(*± *0.00)*
	Crotonaldehyde	µg/L	< LOD	2.844	< LOD	< LOD	< LOD
				*(*± *0.75)*			
	Acrolein	µg/L	0.001	3.98	0.001	0.001	0.001
			(± 0.00)	*(*± *0.45)*	(± 0.00)	(± 0.00)	(± 0.00)
	4-(methylnitrosamino) 1-(3-pyridyl)-1-butanone	ng/L	< LOD	7.838	< LOD	< LOD	< LOD
				*(*± *0.35)*			
	N-Nitrosonornicotine	ng/L	< LOD	8.097	< LOD	< LOD	< LOD
				*(*± *0.48)*			
	Vegetable glycerin	µg/L	< LOD	53.513	577.278	543.559	546.254
				*(*± *6.44)*	*(*± *64.02)*	*(*± *68.29)*	*(*± *74.05)*
	Propylene glycol	µg/L	< LOD	< LOD	179.164	171.293	172.977
					*(*± *21.99)*	*(*± *16.77)*	*(*± *19.66)*
	Guaiacol	ng/L	< LOD	NA	< LOD	< LOD	4.094
							(± 0.18)
	Carbon monoxide	ppm	< LOD	608.398	NA	NA	NA
				*(*± *22.85)*			
	Nicotine	µg/L	< LOD	35.905	< LOD	36.414	36.729
				*(*± *2.78)*		*(*± *2.19)*	*(*± *2.74)*
Aerosol characteristics	Total particulate matter	µg/L	< LOD	574.52	1119.60	1131.36	1112.29
				*(*± *16.58)*	*(*± *73.57)*	*(*± *81.85)*	*(*± *108.56)*
	Mass median aerodynamic diameter	µm	NA	0.815	0.955	0.922	1.012
				*(*± *0.07)*	*(*± *0.10)*	*(*± *0.11)*	*(*± *0.17)*
	Geometric Standard Deviation	NA	NA	1.277	1.393	1.358	1.493
				*(*± *0.07)*	*(*± *0.05)*	*(*± *0.04)*	*(*± *0.10)*

Data are shown as mean ± SD (standard deviation).

LOD for nicotine is 0.03 μg/L

LOD for propylene glycol is 0.75 and 0.73 μg/L for Sham and 3R4F chambers, respectively

LOD for vegetable glycerin is 0.91 μg/L

*CS* cigarette smoke, *LOD* limit of detection, *NA* not analysed

*Guaiacol is a flavor marker in atmosphere

The levels of the tobacco-specific nitrosamines NNK and NNN in the e-vapors were below the limits of detection (LOD), compared to the 7.83 and 8.09 µg/L concentrations, respectively, in 3R4F CS (Table 1). The concentrations of PG and VG were comparable in the three e-vapor aerosols; in contrast, in 3R4F CS, the VG concentration was much lower while the PG concentration was below the LOD (Table 1). Test atmosphere characterization demonstrated that the target concentration for nicotine was met in the 3R4F CS- and the base and test aerosol-exposed groups. Guaiacol, a representative flavor marker, was detected only in the test aerosol group that contained the flavor mix. At the same nicotine concentration, the levels of TPM were higher in the e-vapors than in 3R4F CS, while carbon monoxide was only present in CS. The MMADs and GSDs of the aerosol size distribution were comparable in all aerosols and CS and were all within the respirable range (Asgharian et al. 2014).

### Biomarkers of e-vapor aerosol and 3R4F CS exposure

As expected, the mean urinary levels of SPMA, CEMA, 1-MHBMA, and 2-MHBMA were significantly elevated (*p* < 0.05) in response to CS exposure (185.2, 557, 279.2, and 81.1 ng/mL, respectively) relative to sham exposure (Table 2). The levels of these biomarkers did not differ between the sham group and e-vapor-exposed groups (carrier, base, and test). E-vapor (carrier, base, or test aerosol) exposures led to much lower urinary levels of SPMA (− 99.36%), CEMA (− 99.14%), 1-MHBMA (− 99.61%), and 2-MHBMA (− 98.57%) than CS exposure (*p* < 0.05) (Table 2). Unlike other biomarkers of harmful and potentially harmful constituents, the background levels of HPMA, the acrolein exposure marker, were high in the sham controls, likely because of endogenous production of acrolein (Stevens and Maier 2008). The HPMA concentration in the 3R4F group was twofold higher while those in the e-vapor groups were not statistically different from the sham group.

**Table 2:** Biomarkers of CS and e-vapor aerosol exposure in urine and plasma

	Endpoints	Unit	Time points	Sham	3R4F	CARRIER (PG/VG)	BASE (PG/VG/N)	TEST (PG/VG/N/F)
Marker of exposure in urine (concentration)	SPMA	ng/mL	1M	1.98 (± 0.74)	185.23 (± 37.07)b+	1.83	0.94	0.78
						(± 0.70)	(± 0.47)+	(± 0.19)+
	CEMA	ng/mL	1M	4.78 (± 1.58)	557.03	5.75	3.89	4.68
					(± 118.96)b+	(± 1.50)	(± 1.09)	(± 1.21)
	HPMA	ng/mL	1M	4 330.08 (± 1 633.65)	8,667.75	5,325.63	3,624.28	4,294.58
					(± 1 290.77)b+	(± 1 770.79)	(± 1 224.38)	(± 1 314.31)
	MHBMA1	ng/mL	1M	0.26 (± 0.44)	279.23	1.01	1.46	0.77
					(± 94.73)b+	(± 1.22)	(± 1.52)+	(± 1.10)
	MHBMA2	ng/mL	1M	1.27 (± 0.95)	81.12	1.56	0.78	1.13
					(± 6.98)b+	(± 0.86)	(± 0.75)	(± 0.77)
Marker of Exposure in plasma	Propylene glycol	µg/mL	1M	0.10 (± 0.02)	0.17	3.31	4.37	3.18
					(± 0.06)b+	(± 2.41)+	(± 4.50)+	(± 2.15)+
			4M	0.17 (± 0.11)	0.12	2.13	2.51	1.42
					(± 0.06)b	(± 1.24)+	(± 2.96)+	(± 0.68)+
	Nicotine	ng/mL	1M	0.00 (± 0.00)	236.41	0	132.33	115.01
					(± 247.43)+	(± 0.00)	(± 112.03)+	(± 65.18)+
			4M	0.74 (± 1.54)	99.39	0.3	142.96	83.21
					(± 48.59)+	(± 0.85)	(± 150.44)+	(± 40.49)+
	Cotinine	ng/mL	1M	0	261.95	0	270.23	287.34
				(± 0.00)	(± 105.44)+	(± 0.00)	(± 105.12)+	(± 117.39)+
			4M	0	209.66	0	294.6	287.88
				(± 0.00)	(± 66.41)b+	(± 0.00)	(± 79.71)+	(± 104.74)+
Nicotine metabolites in urine (absolute)	Cotinine	µmol	1M	0	0.03	0	0.04	0.04
				(± 0.00)	(± 0.01)b+	(± 0.00)	(± 0.01)+	(± 0.02)+
			4M	0	0.03	0	0.05	0.05
				(± 0.00)	(± 0.01)b+	(± 0.00)	(± 0.02)+	(± 0.01)+
	Nicotine-1-N- Oxide	µmol	1M	0	0.01	0	0.01	0.01
				(± 0.00)	(± 0.00)+	(± 0.00)	(± 0.00)+	(± 0.00)+
			4M	0	0.02	0	0.02	0.02
				(± 0.00)	(± 0.00)+	(± 0.00)	(± 0.00)+	(± 0.00)+
	Norcotinine	µmol	1M	0	0.01	0	0.02	0.03
				(± 0.00)	(± 0.00)b+	(± 0.00)	(± 0.01)+	(± 0.02)+
			4M	0	0.02	0	0.02	0.02
				(± 0.00)	(± 0.00)+	(± 0.00)	(± 0.00)+	(± 0.00)+
	Nornicotine	µmol	1M	0	0.01	0	0.02	0.02
				(± 0.00)	(± 0.01)b+	(± 0.00)	(± 0.00)+	(± 0.01)+
			4M	0	0.02	0	0.03	0.03
				(± 0.00)	(± 0.01)b+	(± 0.00)	(± 0.01)+	(± 0.00)+
	Total metabolite	µmol	1M	0	0.67	0	0.72	0.81
				(± 0.00)	(± 0.14)+	(± 0.00)	(± 0.20)+	(± 0.42)+
			4M	0	0.67	0	0.8	0.8
				(± 0.00)	(± 0.16)+	(± 0.00)	(± 0.18)+	(± 0.11)+
	Trans-3- hydroxycotinine	µmol	1M	0	0.6	0	0.62	0.71
				(± 0.00)	(± 0.14)+	(± 0.00)	(± 0.19)+	(± 0.38)+
			4M	0	0.59	0	0.68	0.67
				(± 0.00)	(± 0.15)+	(± 0.00)	(± 0.16)+	(± 0.10)+

Value reported as Mean ± SD, n = 8. “+” denotes p < 0.05 versus the sham group; “t” denotes p < 0.05 versus the test group; “c” denotes p < 0.05 versus the carrier group; “b” denotes p < 0.05 versus the base group; n = 8. CS, cigarette smoke. (SPMA, S-phenylmercapturic acid; CEMA, 2-cyanoethylmercapturic acid; 3HPMA, hydroxypropyl mercapturic acid; 1-MHBMA, monohydroxy- butenylmercapturic acid; 2-MHBMA, dihydroxybutylmercapturic acid)

The plasma nicotine and cotinine concentrations in mice exposed to nicotine-containing atmospheres (CS, base and test aerosols) were increased relative to those in the sham and were not different among the groups receiving nicotine-containing aerosols (Table 2). Plasma PG, a biomarker of exposure to e-vapor aerosols, significantly increased in the e-vapor aerosol-exposed groups (carrier, base, and test) compared to the sham control.

### Lung function changes occurred in mice exposed to 3R4F CS but not in mice exposed to e-vapors

**Fig. 1:**
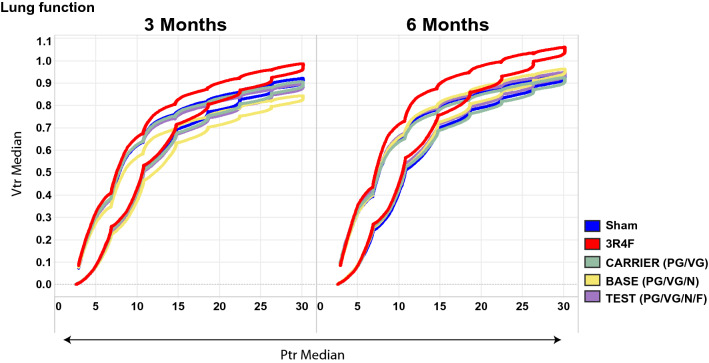
Lung function following exposure to 3R4F CS or e-vapor aerosols. Pressure (Ptr) and volume (Vtr) were recorded at months 3 and 6 to generate pressure–volume (P–V) loops from the medians of replicate measurements. *n* = 12. CS, cigarette smoke

Exposure to CS resulted in an upward and leftward shift of the pressure–volume (P–V) loops in both the inflation and deflation phases of the maneuver (Fig. 1). This corresponded to an increased area enclosed by the P–V loop, higher parameter A and quasi-static compliance (Cst), and lower parameter K and quasi-static elastance in the 3R4F group than in the sham group (Table 3). No consistent differences in lung function parameters were noted between the sham group and any of the e-vapor-exposed groups.

**Table 3:** Lung function following exposure to 3R4F CS or e-vapor aerosol

	Endpoints	Unit	Time points	Sham	3R4F	CARRIER	BASE	TEST
						(PG/VG)	(PG/VG/N)	(PG/VG/N/F)
P–V Loop Area (Hysteresis)	Area	mL/cm H_2_O	3 M	1.73	2.16	1.94	1.85	1.93
				(± 0.26)	(± 0.27)bt +	(± 0.16) +	(± 0.39)	(± 0.26)
			6 M	2.03	2.45	2.08	2.01	2.06
				(± 0.23)	(± 0.36)bt +	(± 0.30)	(± 0.30)	(± 0.24)
P–V Loop Salazar Knowles Equation	A	mL	3 M	0.91	1	0.92	0.87	0.91
				(± 0.05)	(± 0.09)bt +	(± 0.04)	(± 0.09)	(± 0.06)
			6 M	0.91	1.06	0.94	0.95	0.95
				(± 0.09)	(± 0.08)bt +	(± 0.07)	(± 0.07)	(± 0.07)
	K	1/cmH_2_O	3 M	0.14	0.13	0.14	0.14	0.14
				(± 0.01)	(± 0.00)t +	(± 0.01)	(± 0.01)c	(± 0.01)
			6 M	0.14	0.13	0.14	0.14	0.14
				(± 0.01)	(± 0.01)bt +	(± 0.01)	(± 0.00)	(± 0.01)
	Quasi-static compliance	mL/cmH_2_O	3 M	0.09	0.09	0.09	0.08	0.09
				(± 0.01)	(± 0.01)b	(± 0.01)	(± 0.01)c	(± 0.01)
			6 M	0.09	0.1	0.09	0.09	0.09
				(± 0.01)	(± 0.01)b +	(± 0.01)	(± 0.01)	(± 0.01)
	Quasi-static elastance	cmH_2_ O/mL	3 M	11.33	10.9	11.36	12.29	11.46
				(± 0.75)	(± 0.96)b	(± 0.72)	(± 1.33)c +	(± 1.03)
			6 M	11.67	10.36	11.18	11.24	11.14
				(± 1.70)	(± 0.96)b +	(± 0.96)	(± 1.13)	(± 1.11)

The Salazar–Knowles equation and P–V loop area data. Data are presented as mean ± SD n = 12. “+” denotes p < 0.05 versus the sham group; “t” denotes p < 0.05 versus the test group; “c” denotes p < 0.05 versus the carrier group; “b” denotes p < 0.05 versus the base group. *CS* cigarette smoke

### Histopathological changes in mice exposed to 3R4F CS but not in mice exposed to e-vapor aerosols

#### E-vapor aerosol exposure did not induce emphysematous changes in respiratory tract tissues in contrast to 3R4F CS exposure

**Fig. 2:**
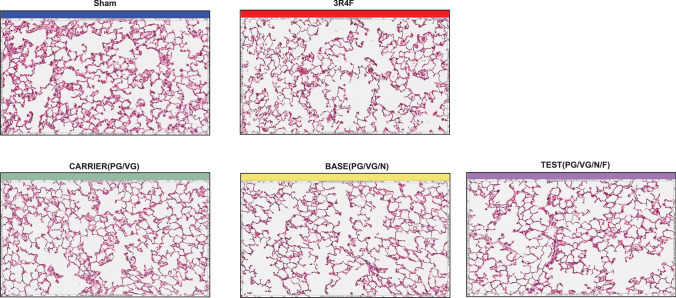
Histopathological evaluation of structural and emphysematous changes in response to 3R4F CS or e-vapor aerosol exposure. Representative images of lung tissue sections stained with hematoxylin and eosin. CS, cigarette smoke

Histopathological findings in the lungs (Fig. 2), expressed as severity scores (Table 4), showed mild to moderate alveolar emphysematous changes in 3R4F CS-exposed mice at months 3 and 6. Minimal emphysematous changes were observed with age in sham and e-vapor-exposed animals. Hyperplasia of the alveolar epithelium was seen at months 3 and 6 in response to 3R4F CS exposure, but not in the sham and e-vapor groups (all groups and time points), and this was statistically significant (*p* < 0.05) (Table 4). The histopathological findings were confirmed by morphometric assessment of emphysema endpoints (Table 4). This assessment demonstrated a significant increase in destructive index (2.8- and 3.1-fold at months 3 and 6, respectively; *p* < 0.05) and mean chord length (12% and 17% at months 3 and 6, respectively; *p* < 0.05) in response to 3R4F CS exposure, relative to sham (Table 4). Emphysematous changes in response to 3R4F CS exposure were also evidenced by significant decreases in the alveolar density in the parenchyma (23% and 26.6% at months 3 and 6, respectively; *p* < 0.05), density of bronchiolar attachments (16.7% and 20.7% at months 3 and 6, respectively; *p* < 0.05), and total number of alveoli (9.7% and 14.4% at months 3 and 6, respectively; *p* < 0.05) as well as by increases in the volume of air (21% and 25% at months 3 and 6, respectively; *p* < 0.05) and total lung volume (16.4% and 19.9% at months 3 and 6, respectively; *p* < 0.05) in CS-exposed mice relative to sham-exposed mice (Table 4; Online Resource 8). These emphysema-related parameters did not differ significantly between the sham group and the e-vapor-exposed groups (carrier, base, and test) after 3 or 6 months of exposure.

**Table 4:** Histopathological and morphological evaluation of structural and emphysematous changes in response to 3R4F cigarette smoke (CS) or e-vapor aerosols exposure

	Endpoints	Unit	Time points	Sham	3R4F	CARRIER	BASE	TEST
						(PG/VG)	(PG/VG/N)	(PG/VG/N/F)
Histopathology respiratory	Emphysema	Score	3 M	0.17	1	0.17	0	0.17
				(± 0.39)	(± 0.60)bt +	(± 0.39)	(± 0.00)	(± 0.39)
			6 M	0.25	1.83	0.17	0.17	0.17
				(± 0.45)	(± 0.94)bt +	(± 0.39)	(± 0.39)	(± 0.39)
	Alveolar epithelium, hyperplasia	Score	3 M	0	0.58	0	0	0
				(± 0.00)	(± 0.51)bt +	(± 0.00)	(± 0.00)	(± 0.00)
			6 M	0	0.67	0	0	0
				(± 0.00)	(± 0.65)bt +	(± 0.00)	(± 0.00)	(± 0.00)
Morphometry	Mean cord length	µm	3 M	47.17	52.86	46.82	46.65	46.77
				(± 3.85)	(± 4.84)bt +	(± 2.35)	(± 3.96)	(± 3.23)
			6 M	44.1	51.58	43.78	45.15	45.04
				(± 3.52)	(± 5.49)bt +	(± 2.76)	(± 3.55)	(± 2.82)
	Destructive index	%	3 M	10.6	30.75	13.28	10.4	10.39
				(± 4.84)	(± 7.22)bt +	(± 4.82)	(± 3.42)	(± 4.37)
			6 M	8.59	26.94	8.48	7.49	8.41
				(± 3.75)	(± 8.37)bt +	(± 2.29)	(± 1.86)	(± 2.94)
	Bronchiolar attachments	mm −1	3 M	14.78	12.31	14.6	14.78	15.01
				(± 1.60)	(± 1.43)bt +	(± 1.01)	(± 1.61)	(± 1.51)
			6 M	14.19	11.25	13.42	13.8	14.27
				(± 0.97)	(± 2.41)bt +	(± 1.40)	(± 1.04)	(± 1.43)
	Number density of alveoli in parenchyma	106/L	3 M	3,387.09	2,609.67	3,454.18	3,452.33	3,481.50
				(± 217.07)	(± 229.94)bt +	(± 314.77)	(± 290.52)	(± 455.13)
			6 M	3,198.17	2,346.08	3,092.73	3,137.08	3,079.82
				(± 417.06)	(± 343.99)bt +	(± 261.73)	(± 288.14)	(± 274.24)
	Total lung volume	μL	3 M	80.93	94.21	82.49	81.04	80.9
				(± 7.46)	(± 10.55)bt +	(± 9.44)	(± 13.63)	(± 9.99)
			6 M	75.91	90.98	80.22	80.96	80.49
				(± 10.34)	(± 11.85)b +	(± 14.59)	(± 10.48)	(± 16.04)
	Total number of alveoli		3 M	2,29,104.20	2,06,764.17	2,46,898.40	2,42,111.90	2,30,627.82
				(± 26 994.84)	(± 35 729.69)b	(± 30 385.81)	(± 37 367.89)	(± 32 820.23)
			6 M	2,06,346.92	1,76,445.92	2,11,185.73	2,10,411.00	2,09,097.00
				(± 37 335.51)	(± 23 919.82)b +	(± 43 445.02)	(± 36 120.51)	(± 47 599.10)
	Total volume of air	μL	3 M	56.66	68.56	59.82	58.35	55.71
				(± 5.01)	(± 8.76)bt +	(± 5.49)	(± 9.81)	(± 8.08)
			6 M	53.41	66.78	56.67	56.44	56.38
				(± 7.53)	(± 9.86)bt +	(± 11.58)	(± 8.70)	(± 11.41)

Lung tissues were sectioned and stained with hematoxylin and eosin. Data are presented as mean ± SD; n = 10–12. “+” denotes p < 0.05 versus the sham group; “t” denotes p < 0.05 versus the test group; “b” denotes p < 0.05 versus the base group. The histopathological score definitions were: (1) minimal; (2) mild; (3) moderate; (4) marked; and (5) severe. *CS* cigarette smoke

#### E-vapor exposure induced fewer histopathological changes in the upper respiratory tract than 3R4F CS

Histopathological assessment of the nose tissue revealed that 3R4F CS exposure for up to 6 months induced respiratory epithelial hyperplasia and respiratory epithelial squamous metaplasia at the entrance of the nose (level 1) (Table 5). Additionally, CS-exposed mice showed a significant increase in severity of olfactory epithelium atrophy (nose level 2) at month 6 compared to sham mice (Table 5). The aerosol-exposed groups (carrier, base, and test) did not show significant changes relative to the sham group. There were no signs of inflammatory cell infiltration in the nose tissue in any of the aerosol-exposed groups. At nose level 3, in comparison with sham, aerosol exposure (carrier, base, and test groups) induced a significant increase in the extent of eosinophilic globules in the olfactory epithelium at months 3 and 6 (*p* < 0.05); the intra-animal variability was high, as reflected by the high standard deviations of these mean scores. A similar increase in the severity score for eosinophilic globules was also observed at nose level 4, in the olfactory epithelium, in e-vapor-exposed mice (carrier, base, and test) (Table 5).

**Table 5:** Histopathological findings in nasal epithelial tissues observed in response to 3R4F CS or e-vapor exposure

	Endpoints	Unit	Time points	Sham	3R4F	CARRIER (PG/VG)	BASE (PG/VG/N)	TEST (PG/VG/N/F)
Nose level 1	Respiratory epithelium, degeneration	Score	3 M	0.09	0	0.25	0.08	0.17
				(± 0.30)	(± 0.00)	(± 0.45)	(± 0.29)	(± 0.39)
			6 M	0.08	0	0	0.08	0
				(± 0.29)	(± 0.00)	(± 0.00)	(± 0.29)	(± 0.00)
	Respiratory epithelium, eosinophilic globules	Score	3 M	1.09	0.08	0.58	0.92	0.92
				(± 1.14)	(± 0.29)bt +	(± 0.67)	(± 0.90)	(± 0.90)
			6 M	0.33	0.17	0.33	0.33	0.42
				(± 0.78)	(± 0.39)	(± 0.65)	(± 0.49)	(± 0.79)
	Respiratory epithelium, hyperplasia	Score	3 M	0.45	3.58	0.58	0.5	0.42
				(± 0.52)	(± 0.51)bt +	(± 0.67)	(± 0.67)	(± 0.51)
			6 M	0.58	3.58	0.5	0.33	0.33
				(± 0.90)	(± 0.51)bt +	(± 0.67)	(± 0.49)	(± 0.49)
	Respiratory epithelium, lamina propria, inflammatory cell infiltration	Score	3 M	0	0	0	0	0
				(± 0.00)	(± 0.00)	(± 0.00)	(± 0.00)	(± 0.00)
			6 M	0.08	0	0	0	0
				(± 0.29)	(± 0.00)	(± 0.00)	(± 0.00)	(± 0.00)
	Respiratory epithelium, squamous epithelial metaplasia	Score	3 M	0	2.75	0	0	0
				(± 0.00)	(± 0.45)bt +	(± 0.00)	(± 0.00)	(± 0.00)
			6 M	0	2.83	0	0	0
				(± 0.00)	(± 0.39)bt +	(± 0.00)	(± 0.00)	(± 0.00)
	Respiratory epithelium,	Score	3 M	0	0	0	0	0
	sub-epithelial blood vessels, dilatation			(± 0.00)	(± 0.00)	(± 0.00)	(± 0.00)	(± 0.00)
			6 M	0.17	0	0	0	0
				(± 0.58)	(± 0.00)	(± 0.00)	(± 0.00)	(± 0.00)
	Respiratory region, hyperplasia of goblet cells, septum	Score	3 M	0.09	0	0.08	0.17	0.08
				(± 0.30)	(± 0.00)	(± 0.29)	(± 0.39)	(± 0.29)
			6 M	0.5	0.17	0	0.17	0.33
				(± 0.90)	(± 0.39)	(± 0.00) +	(± 0.39)	(± 0.65)
	Respiratory region, lumen, amorphous eosinophilic material	Score	3 M	0	0	0.08	0	0
				(± 0.00)	(± 0.00)	(± 0.29)	(± 0.00)	(± 0.00)
			6 M	0	0	0	0	0
				(± 0.00)	(± 0.00)	(± 0.00)	(± 0.00)	(± 0.00)
	Respiratory region, lumen, cell debris	Score	3 M	0	0.08	0	0	0.17
				(± 0.00)	(± 0.29)	(± 0.00)	(± 0.00)	(± 0.58)
			6 M	0.08	0	0	0	0
				(± 0.29)	(± 0.00)	(± 0.00)	(± 0.00)	(± 0.00)
	Respiratory region, lumen, foreign material	Score	3 M	0	0.08	0.08	0	0.17
				(± 0.00)	(± 0.29)	(± 0.29)	(± 0.00)	(± 0.58)
			6 M	0	0	0	0	0
				(± 0.00)	(± 0.00)	(± 0.00)	(± 0.00)	(± 0.00)
	Respiratory region, lumen, red blood cells	Score	3 M	0	0	0	0	0
				(± 0.00)	(± 0.00)	(± 0.00)	(± 0.00)	(± 0.00)
			6 M	0.08	0	0	0	0
				(± 0.29)	(± 0.00)	(± 0.00)	(± 0.00)	(± 0.00)
Nose level 2	Olfactory epithelium, atrophy	Score	3 M	0.17	0.58	0.25	0.17	0.17
				(± 0.39)	(± 0.79)	(± 0.45)	(± 0.39)	(± 0.39)
			6 M	0	0.5	0	0	0
				(± 0.00)	(± 0.80)bt +	(± 0.00)	(± 0.00)	(± 0.00)
	Olfactory epithelium, lamina propria, loss of nerve bundles	Score	3 M	0	0	0	0	0
				(± 0.00)	(± 0.00)	(± 0.00)	(± 0.00)	(± 0.00)
			6 M	0	0.25	0	0	0
				(± 0.00)	(± 0.87)	(± 0.00)	(± 0.00)	(± 0.00)
	Olfactory epithelium, lamina propria, lymphocytic cell aggregates	Score	3 M	0	0	0	0	0
				(± 0.00)	(± 0.00)	(± 0.00)	(± 0.00)	(± 0.00)
			6 M	0.25	0	0	0	0
				(± 0.87)	(± 0.00)	(± 0.00)	(± 0.00)	(± 0.00)
	Respiratory epithelium, eosinophilic globules	Score	3 M	0.33	0.33	0.08	0.08	0
				(± 0.78)	(± 0.65)	(± 0.29)	(± 0.29)	(± 0.00)
			6 M	0	0	0.08	0	0
				(± 0.00)	(± 0.00)	(± 0.29)	(± 0.00)	(± 0.00)
	Respiratory epithelium, hyperplasia	Score	3 M	0	0.25	0	0	0
				(± 0.00)	(± 0.45)	(± 0.00)	(± 0.00)	(± 0.00)
			6 M	0	0.17	0	0	0
				(± 0.00)	(± 0.39)	(± 0.00)	(± 0.00)	(± 0.00)
	Respiratory epithelium,	Score	3 M	0	0.08	0	0	0
	sub-mucosal gland, ectasia			(± 0.00)	(± 0.29)	(± 0.00)	(± 0.00)	(± 0.00)
			6 M	0.08	0	0	0	0
				(± 0.29)	(± 0.00)	(± 0.00)	(± 0.00)	(± 0.00)
	Respiratory region, lumen, cell debris	Score	3 M	0	0	0	0	0
				(± 0.00)	(± 0.00)	(± 0.00)	(± 0.00)	(± 0.00)
			6 M	0.08	0	0	0.17	0
				(± 0.29)	(± 0.00)	(± 0.00)	(± 0.58)	(± 0.00)
	Respiratory region, lumen, foreign material	Score	3 M	0	0	0	0.17	0.25
				(± 0.00)	(± 0.00)	(± 0.00)	(± 0.58)	(± 0.62)
			6 M	0	0	0	0.25	0.08
				(± 0.00)	(± 0.00)	(± 0.00)	(± 0.62)	(± 0.29)
Nose level 3	Olfactory epithelium, atrophy	Score	3 M	0	0	0	0	0
				(± 0.00)	(± 0.00)	(± 0.00)	(± 0.00)	(± 0.00)
			6 M	0	0.08	0	0	0
				(± 0.00)	(± 0.29)	(± 0.00)	(± 0.00)	(± 0.00)
	Olfactory epithelium, eosinophilic globules	Score	3 M	0.42	0.67	1.83	1.92	1.83
				(± 0.67)	(± 0.78)bt	(± 1.47) +	(± 1.16) +	(± 1.27) +
			6 M	0.25	0.92	2.75	1.83	2.17
				(± 0.45)	(± 0.90)bt +	(± 0.75) +	(± 0.94)c +	(± 1.11) +
Nose level 4	Olfactory epithelium, eosinophilic globules	Score	3 M	0	0.25	1.18	2	1.75
				(± 0.00)	(± 0.87)bt	(± 1.25) +	(± 1.35) +	(± 1.36) +
			6 M	0.25	0.33	1.17	1	1
				(± 0.87)	(± 0.89)bt	(± 0.83) +	(± 0.85) +	(± 0.74) +
	Pharyngeal duct, epithelium, eosinophilic globules	Score	3 M	0	0.17	0	0	0
				(± 0.00)	(± 0.58)	(± 0.00)	(± 0.00)	(± 0.00)
			6 M	0	0	0	0	0
				(± 0.00)	(± 0.00)	(± 0.00)	(± 0.00)	(± 0.00)
	Sub-mucosal gland, ectasis	Score	3 M	0.08	0	0	0	0
				(± 0.29)	(± 0.00)	(± 0.00)	(± 0.00)	(± 0.00)
			6 M	0	0	0	0	0
				(± 0.00)	(± 0.00)	(± 0.00)	(± 0.00)	(± 0.00)

Data are presented as mean ± SD; n = 11–12. “+” denotes p < 0.05 versus the sham group; “t” denotes p < 0.05 versus the test group; “b” denotes p < 0.05 versus the base group. *CS* cigarette smoke

In the larynx, exposure to CS caused the typical moderate to marked epithelial hyperplasia and squamous metaplasia at the vocal cords, base of the epiglottis, and floor of the larynx (Online Resource 10) at both time points. In contrast, no changes at the vocal cords and floor of the larynx relative to sham were observed following exposure to the aerosols; at the base of the epiglottis, minimal squamous metaplasia was seen in the carrier group at the 3-month time point, and minimal to mild squamous metaplasia was observed in the test group at the 6-month time point. However, these laryngeal changes in the aerosol-exposed groups were significantly lower (*p* < 0.05) when compared with the lesions in the CS group. In the trachea, no effects were observed in response to CS or aerosol exposure (Online Resource 9).

### E-vapor exposure induced less inflammatory cell infiltration in the lungs than 3R4F CS

Histopathological evaluation was performed to assess lung inflammation. There was a significant increase in the extent of macrophage (unpigmented, yellow-pigmented, and pigmented macrophage nests) and neutrophilic granulocyte infiltrates in response to 3R4F CS exposure at 3 and 6 months (*p* < 0.05; Table 6). In contrast, e-vapor exposure did not result in increased immune cell infiltrates in the lungs relative to sham exposure either at 3 or 6 months. Additionally, 3R4F CS exposure, but not e-vapor aerosol exposure, for 6 months caused significant increases in the extent of alveolar interstitium/sub-pleural lymphocytic cell aggregates and lymphocytes/plasma cell infiltrates in the alveolar lumen (Table 6). The increase in inflammatory cells in the lungs was also associated with a significant increase in absolute and relative lung weights in response to CS exposure. In contrast, aerosol exposure did not result in lung weight changes (Online Resource 10).

**Table 6:** Histopathological evaluation of inflammatory changes in response to 3R4F cigarette smoke (CS) or e-vapors exposure

	Endpoints	Unit	Time points	Sham	3R4F	CARRIER (PG/VG)	BASE (PG/VG/N)	TEST (PG/VG/N/F)
Histpathology in Lung	Alveolar lumen, unpigmented macrophages	Score	3 M	0.17 (± 0.39)	2.92 (± 0.29)bt +	0.25 (± 0.45)	0.33 (± 0.49)	0.25 (± 0.45)
			6 M	0.08 (± 0.29)	2.67 (± 0.65)bt +	0.17 (± 0.39)	0.17 (± 0.39)	0.33 (± 0.49)
	Alveolar lumen, yellow pigmented macrophages	Score	3 M	0.00 (± 0.00)	2.25 (± 0.97)bt +	0.00 (± 0.00)	0.00 (± 0.00)	0.00 (± 0.00)
			6 M	0.00 (± 0.00)	2.92 (± 1.31)bt +	0.00 (± 0.00)	0.00 (± 0.00)	0.00 (± 0.00)
	Alveolar lumen, pigmented macrophage nests	Score	3 M	0.00 (± 0.00)	0.83 (± 0.58)bt +	0.00 (± 0.00)	0.00 (± 0.00)	0.00 (± 0.00)
			6 M	0.00 (± 0.00)	1.33 (± 0.98)bt +	0.00 (± 0.00)	0.00 (± 0.00)	0.00 (± 0.00)
	Alveolar lumen, neutrophilic granulocytes	Score	3 M	0.00 (± 0.00)	0.92 (± 0.29)bt +	0.08 (± 0.29)	0.00 (± 0.00)	0.00 (± 0.00)
			6 M	0.00 (± 0.00)	0.58 (± 0.51)bt +	0.00 (± 0.00)	0.00 (± 0.00)	0.00 (± 0.00)
	Alveolar interstitium/ sub-pleural, lymphocytic cell aggregates	Score	3 M	0.00 (± 0.00)	0.00 (± 0.00)	0.00 (± 0.00)	0.25 (± 0.87)	0.00 (± 0.00)
			6 M	0.00 (± 0.00)	1.25 (± 1.06)bt +	0.00 (± 0.00)	0.08 (± 0.29)	0.00 (± 0.00)
	Alveolar lumen, lymphocytes/plasma cells	Score	3 M	0.08 (± 0.29)	0.08 (± 0.29)	0.00 (± 0.00)	0.00 (± 0.00)	0.00 (± 0.00)
			6 M	0.00 (± 0.00)	0.42 (± 0.51) +	0.00 (± 0.00)	0.08 (± 0.29)	0.08 (± 0.29)

Lung tissues were sectioned and stained with hematoxylin and eosin. Data are presented as mean ± SD; n = 10–12. “+” denotes p < 0.05 versus the sham group; “t” denotes p < 0.05 versus the test group; “b” denotes p < 0.05 versus the base group. The histopathological score definitions were: (1) minimal; (2) mild; (3) moderate; (4) marked; and (5) severe. *CS* cigarette smoke

Relative to the sham exposure, 3R4F CS exposure significantly increased the total number of free lung cells in BALF (a 4.3-fold increase at month 3 and 2.4-fold increase at month 6; *p* < 0.05; (Table 7). Analysis of the inflammatory cell subpopulations demonstrated a significant increase in the numbers of dendritic cells, lymphocytes, macrophages, and neutrophils in 3R4F CS-exposed mice relative to sham mice (*p* < 0.05; Table 7). The elevated lymphocyte number in BALF in the 3R4F CS group was associated with increases in B-cell and CD4 + and CD8 + T-lymphocyte numbers (Table 7). In comparison with 3R4F CS-exposed mice, e-vapor-exposed mice (carrier, base and test) showed significantly lower total numbers of free lung cells at months 3 (− 72.4%, − 80.1%, − 74.3%) and 6 (− 66.2%, − 69.3%, and − 61.6%), respectively; *p* < 0.05 for all comparisons; (Table 7). The aerosol-exposed groups (carrier, base, and test) did not show significant increases in inflammatory cell influx into the lung lumen relative to the sham group.

**Table 7:** Immune cells in BALF in response to 3R4F CS or e-vapor aerosol exposure

	Endpoints	Time points	Sham	3R4F	CARRIER (PG/VG)	BASE (PG/VG/N)	TEST (PG/VG/N/F)
Free lung cells collection	Total cells	3 M	334′188	1′448′743	399′541	287′214	371′483
			(± 95 566.84)	(± 988 432.46)bt +	(± 137 053.79)	(± 67 032.76)c	(± 101 221.45)b
		6 M	474′082	1′183′290	399′430	362′200	453′384
			(± 134 830.77)	(± 751 019.47)bt +	(± 109 653.26)	(± 79 690.68) +	(± 208 723.28)
Free lung cells differentiation count	Dendritic cell counts	3 M	3′034	84′279	4′021	1′739	3′128
			(± 1 204.68)	(± 74 326.54)bt +	(± 5 233.39)	(± 658.07) +	(± 4 379.27)
		6 M	5′577	44′145	5′607	3′710	5′461
			(± 2 867.20)	(± 32 459.76)bt +	(± 6 508.64)	(± 2 617.98)	(± 6 405.46)
	Leukocyte counts	3 M	316′220	1′222′819	381′461	269′305	348′447
			(± 89 670.28)	(± 871 870.70)bt +	(± 130 870.87)	(± 62 435.11)c	(± 101 011.80)
		6 M	447′820	1′017′674	374′090	343′356	428′558
			(± 125 517.10)	(± 647 201.06)bt +	(± 109 713.75)	(± 74 701.31) +	(± 204 039.23)
	Lymphocyte count	3 M	4′034	133′886	11′589	2′980	7′374
			(± 1 578.15)	(± 100 085.33)bt +	(± 28 626.86)	(± 1 974.65)	(± 16 511.54)
		6 M	17′139	195′865	21′409	7′140	28′461
			(± 21 689.10)	(± 186 098.48)bt +	(± 45 502.80)	(± 4 957.89)	(± 74 512.42)
	Macrophage counts	3 M	303′141	553′521	357′738	260′346	332′647
			(± 86 433.49)	(± 324 504.26)b +	(± 104 055.12)	(± 61 655.63)c	(± 83 763.64)b
		6 M	409′223	402′741	335′358	323′289	377′863
			(± 95 566.71)	(± 177 533.82)	(± 62 497.75)	(± 64 402.20) +	(± 116 000.23)
	Neutrophil count	3 M	428	370′029	965	156	171
			(± 343.76)	(± 349 199.59)bt +	(± 2 320.29)	(± 51.84) +	(± 119.07) +
		6 M	3′749	301′882	1′081	594	4′345
			(± 7 534.35)	(± 218 499.21)bt +	(± 1 238.78)	(± 278.06) +	(± 11 181.91)
Free lung cells lymphocytes differentiation count		3 M	168	20′338	822	81	167
	B-Lymphocyte count		(± 311.27)	(± 22 446.32)bt +	(± 2 428.04)	(± 121.17)	(± 472.80)
		6 M	1′216	22′774	1′219	313	1′683
			(± 2 711.40)	(± 33 815.90)bt +	(± 3 209.35)	(± 346.45)	(± 4 944.51)
	T-Lymphocyte count	3 M	1′995	98′973	5′589	1′448	1′482
			(± 2 508.29)	(± 72 267.86)bt +	(± 15 123.37)	(± 2 108.38)	(± 2 310.27)
		6 M	6′111	104′191	7′533	2′433	9′815
			(± 8 013.10)	(± 101 830.93)bt +	(± 16 260.42)	(± 1 668.11)	(± 25 950.24)
		3 M	517	24′416	2′042	483	507
	CD4 +		(± 840.98)	(± 16 443.08)bt +	(± 5 891.25)	(± 881.70)	(± 867.37)
	T-Lymphocyte count	6 M	1′990	29′925	2′872	756	5′048
			(± 2 533.40)	(± 31 173.02)bt +	(± 6 441.27)	(± 619.39)	(± 14 251.45)
	CD8 +	3 M	290	13′579	185	211	120
	T-Lymphocyte count		(± 666.63)	(± 11 925.45)bt +	(± 317.80)	(± 456.97)	(± 201.73)
		6 M	836	15′522	1′427	234	1′023
			(± 1 322.04)	(± 15 334.99)bt +	(± 3 473.60)	(± 161.13)	(± 2 710.38)

Immune cell subtypes were identified and quantified by flow cytometry. Absolute inflammatory cell counts and lymphocyte differentiation are presented as mean ± SD; n = 10. “+” denotes p < 0.05 versus the sham group; “t” denotes p < 0.05 versus the test group; “c” denotes p < 0.05 versus the carrier group; “b” denotes p < 0.05 versus the base group. *BALF* bronchoalveolar lavage fluid

#### E-vapor exposure induced fewer changes in inflammatory mediator levels in BALF than 3R4F CS

**Fig. 3:**
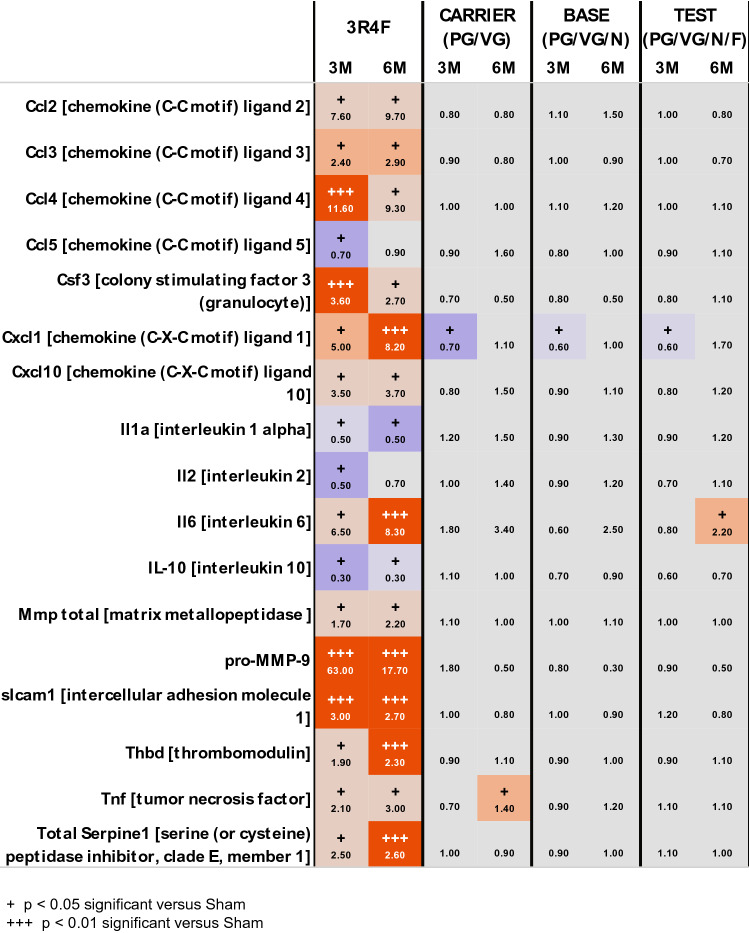
Inflammatory mediators in BALF in response to 3R4F CS or e-vapor aerosol exposure. Inflammatory mediators in BALF. Cell-free BALF supernatants were analyzed using a multiplexed bead array. The ratio of inflammatory mediators is given as the median of levels in treated mice over the median of levels in sham-exposed mice at the same time point (see color scale). Analytes with statistically significant differences at least in one comparison with the sham group are shown. “ + ” denotes *p* < 0.05; “ +  +  + ” denotes *p* < 0.001; *n* = 10. Orange shades indicate significantly elevated analytes, and blue shades indicate significantly decreased analytes. The full list of analytes is provided on the INTERVALS website (10.26126/intervals.8lafdu.1). *BALF* bronchoalveolar lavage fluid

Consistent with the increased number of inflammatory cells in BALF, exposure to 3R4F CS induced a significant increase in the abundance of inflammatory mediators after 3 and 6 months (Fig. 3). In particular, the BALF concentrations of chemokines Ccl2 [chemokine (C–C motif) ligand 2], Ccl3, Ccl4, Cxcl10 [chemokine (C-X-C motif) ligand 10], and Cxcl1 increased, as did those of Icam1 (intercellular adhesion molecule 1), Tnf (tumor necrosis factor), Il6 (interleukin 6), Serpine1 [serine (or cysteine) peptidase inhibitor, clade E, member 1], pro-Mmp9 (matrix metallopeptidase 9), total Mmp (matrix metallopeptidase), and Thbd (thrombomodulin) (Fig. 3). E-vapor aerosol  exposure resulted in fewer changes in inflammatory mediators in BALF. After 3 months of exposure, Cxcl1 levels were significantly lower in the carrier, base, and test groups than in the sham group (*p* < 0.05). The levels of Tnf and Il6 increased slightly but significantly (*p* < 0.05) following 6 months of exposure to base and test aerosols, respectively.

#### E-vapor exposure induced lower Mmp activity in BALF than 3R4F CS

The gelatinolytic Mmp activity in BALF in 3R4F CS-exposed mice was 1.7- and 2.2-fold higher at months 3 and 6, respectively (*p* < 0.05), than that in the sham- and aerosol-exposed mice. The Mmp activity in BALF in all e-vapor groups was close to that in the sham group and significantly lower than that in the 3R4F CS group (*p* < 0.05; Table 8).

**Table 8:** Mmp activity in BALF

Endpoint	Unit	Time points	Sham	3R4F	CARRIER	BASE	TEST
					(PG/VG)	(PG/VG/N)	(PG/VG/N/F)
MMP total	(U/L)	3 M	1.16 (± 0.40)	1.99 (± 0.62)bt +	1.31 (± 0.64)	1.16 (± 0.40)	1.14 (± 0.32)
		6 M	0.81 (± 0.29)	1.82 (± 0.90)bt +	0.78 (± 0.26)	0.87 (± 0.48)	0.83 (± 0.21)

Matrix metalloproteinase (Mmp) activity in BALF. Mmp activity is represented as mean ± SD; n = 10. “+” denotes p < 0.05 versus the sham group; “t” denotes p < 0.05 versus the test group; “b” denotes p < 0.05 versus the base group

### E-vapor exposure causes less molecular dysregulation in the respiratory tract than 3R4F CS exposure

Exposure to CS triggered molecular dysregulation in lung tissues. Analysis of nasal and tracheal tissues revealed that exposure to 3R4F CS also triggered molecular dysregulation in the upper respiratory tract and differentially altered the expression levels of 134 and 186 genes in respiratory nasal epithelium and the trachea, respectively, after 6 months (Online Resource 11ad). No significant gene expression changes were observed in response to e-vapor exposure.

**Fig. 4:**
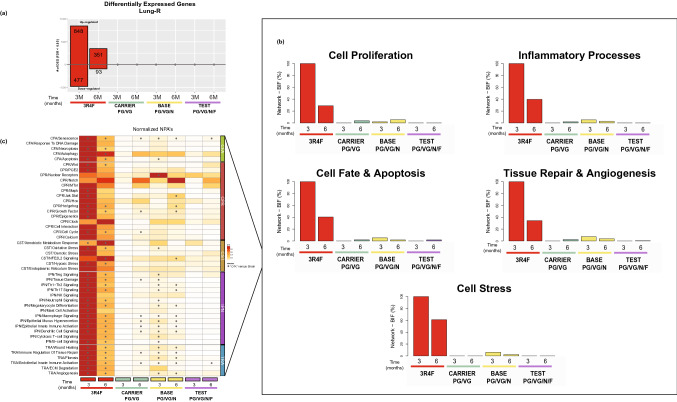
Systems toxicology analysis of dysregulated genes in the lungs. **a** Number of differentially expressed genes. **b** RBIF for treatment versus sham. The percentages show the RBIF, which is derived from the cumulated network perturbations caused by the treatment relative to the reference, defined as the treatment comparison showing the highest perturbation (i.e., at the 6-month time point). **c** Heatmap of NPA scores summarizing subnetwork NPAs relative to the maximum NPA in each subnetwork. Stars indicate significant perturbations: A network is considered perturbed if, in addition to the significance of the NPA score with respect to the experimental variation, the two companion statistics (O and K), derived to inform on the specificity of the NPA score with respect to the biology described in the network, are significant. *O and K statistic *p* values < 0.05 and significant with respect to the experimental variation; *n* = 8–10. *RBIF* relative biological impact factor; *NPA* network perturbation amplitude

In lung tissues, in comparison with sham exposure, CS exposure dysregulated 1325 genes at month 3 and 444 genes at month 6 (Fig. 4a). E-vapor exposure did not alter gene expression significantly at either time point. The causal biological network enrichment approach, based on transcriptomics data from the lungs, showed the highest impact (expressed as the BIF) in 3R4F CS-exposed mice at both months 3 and 6 (Fig. 4b). The relative BIF (with the CS BIF set at 100%) for the e-vapor-exposed groups remained close to zero. Investigation of the underlying network categories demonstrated that mechanisms related to inflammatory responses, cell stress responses, cell fate and apoptosis, cell proliferation, and tissue repair and angiogenesis contributed significantly to the perturbations caused by 3R4F CS exposure (Fig. 4b). At matched nicotine levels, aerosol exposure (carrier, base, and test) caused less network perturbation, suggesting smaller impact on the lung transcriptome than 3R4F CS exposure (Fig. 4b and c).

#### No significant changes in the lung lipidome and proteome were observed in response to e-vapor exposure

**Fig. 5:**
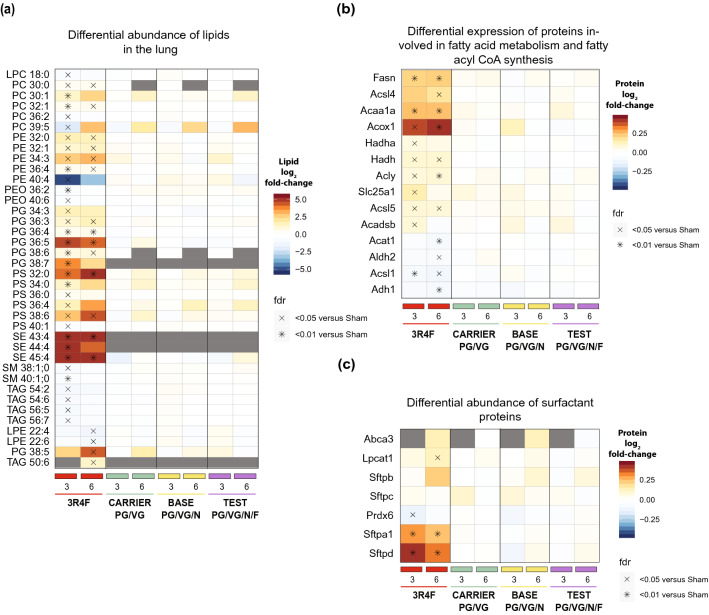
Systems toxicology analysis of lipids and proteins in the lungs. **a** Differential abundance of lipids in the lungs; *n* = 10. Log_2_ fold changes versus sham are color-coded, and statistical significance is indicated. *FDR-adjusted *p* < 0.01; ^X^FDR-adjusted *p* < 0.05. Only lipids with significant differential abundance in any contrast are shown. **b** Differential expression of proteins involved in fatty acid metabolism and fatty acyl coenzyme A (CoA) synthesis (as defined by the KEGG and Reactome databases). Only proteins with significant differential abundance in any contrast are shown. **c** Differential abundance of surfactant proteins. *PC* phosphatidylcholine; *PG* phosphatidylglycerol. *FDR* false discovery rate; *KEGG* Kyoto Encyclopedia of Genes and Genomes

3R4F CS exposure affected the lung proteome and lipidome (Online Resource 12). Specifically, CS exposure broadly affected several lipid classes, including glycerolipids (such as triacylglycerols) and glycerophospholipids (such as phosphatidylcholine, phosphatidylethanolamine, phosphatidylglycerol, and phosphatidylserine) (Fig. 5a). At the same time, CS exposure increased the abundance of proteins such as fatty acid synthase (Fasn), the rate-limiting enzyme for fatty acid synthesis (Fig. 5b). The abundances of several degradation enzymes of fatty acids (Hadha, Acaa1a, Acadsb, and Acox1) were also increased. Such effects in the aerosol-exposed groups were not statistically significant relative to the sham group (Fig. 5a–c).

Surfactant metabolism was clearly affected by 3R4F CS exposure and involved both surfactant proteins and surfactant-associated lipids. In particular, CS exposure upregulated the expression of surfactant proteins Sftpa1 (pulmonary surfactant-associated protein A1) and Sftpd (pulmonary surfactant-associated protein D), which are known to regulate immune defense and surfactant homeostasis (Fig. 5c) (Whitsett et al. 2015). In contrast, the structural surfactant proteins Sftpb (pulmonary surfactant-associated protein B) and Sftpc (pulmonary surfactant-associated protein C) were not significantly affected by CS exposure. After 6 months of CS exposure, the levels of lysophosphatidylcholine acyltransferase (Lpcat1), a surfactant lipid synthesis enzyme, also increased. Additionally, CS exposure also increased the abundance of candidate surfactant lipids such as PC 32:1, PC 30:1, and PC 30:0 (Fig. 5a). Altogether, 3R4F CS exposure resulted in broad perturbation of the lung lipidome and lung lipid metabolism, including surfactant proteins and surfactant-associated lipids. In contrast, none of the aerosols exerted a significant effect on the lung lipidome or surfactants.

#### E-vapor exposure had a smaller impact on DNA methylation of lung transcriptional enhancers than 3R4F CS

Very few promoters (74 out of 23,783; 0.3%) exhibited significant changes in DNA methylation (Online Resource 13). Among the candidate enhancers (low-methylated regions), 1379 elements out of 121,285 (1.14%) exhibited significant changes in DNA methylation in at least one comparison (Online Resource 14a). At both time points, 3R4F CS exposure was associated with the highest number of differentially methylated enhancers, the majority of which were hypermethylated (Online Resource 14b). Aerosols from the carrier, base, and test formulations affected the methylation of much fewer enhancers.

## Discussion

The objective of this study was to assess the impact of exposure to e-liquid aerosols from a capillary aerosol generator on the respiratory system and COPD-related endpoints and compare it to the impact of exposure to CS and fresh air. According to our exposure regimen and the body surface area conversion factor of 12.3—assuming a 0.03 L/min minute volume, a 25 g body weight, and complete retention of nicotine in mice—the estimated delivered dose was 193 µg nicotine per day, which corresponds to a human equivalent nicotine dose of 37.5 mg/day (equivalent to 20 cig/day if we consider 1.8 mg of nicotine per cigarette) (CDER 2005).

3R4F CS exposure, but not e-vapor aerosol exposure, caused a sustained inflammatory response in the lungs, as evidenced by the histopathological findings, pronounced immune cell infiltration and inflammatory mediator secretion. Sustained inflammation is the key hallmark that drives the pathophysiological changes observed in COPD (Botelho et al. 2010; D'Hulst A et al. 2005; Sharafkhaneh et al. 2008). Infiltrating immune cells, in particular alveolar macrophages and neutrophils, secrete not only pro-inflammatory cytokines and growth factors but also a variety of elastolytic enzymes, including neutrophil elastase, Mmp2, Mmp9, Mmp12, and cathepsin K, L, and S, which damage lung parenchyma and cause emphysematous changes (Barnes et al. 2003; Parks and Shapiro 2001; Sandhaus and Turino 2013). Similar to previous studies (Phillips et al. 2019, 2016, 2015; Rangasamy et al. 2009), the present analyses confirm a sustained inflammatory response in 3R4F CS-exposed animals and highlight a local milieu prone to structural changes (Churg et al. 2004; Ohnishi et al. 1998; Shapiro et al. 2003). At similar levels of nicotine, e-vapor exposure resulted in substantially lower lung inflammation and did not cause notable changes in inflammatory mediator levels or MMP activity. Our systems toxicology endpoints—assessing the holistic effects of CS and aerosol exposure on the lung transcriptome, proteome, lipidome, and DNA methylation—consistently support the lower biological impacts of e-vapor aerosols compared to 3R4F CS.

CS exposure has been associated with altered DNA methylation patterns in multiple tissues in both humans and mice (Choukrallah et al. 2019a, 2019b; Phillips et al. 2019; Shenker et al. 2013). In line with these reports, we found that CS exposure was mainly associated with hypermethylation of candidate enhancers in the lungs. E-vapor aerosols  exposure affected a much smaller number of loci than CS exposure suggesting that all e-vapor aerosols (carrier, base, or test) have a lesser impact on DNA than 3R4F CS.

The transcriptomic analysis demonstrated activation of inflammatory and oxidative stress mechanisms in respiratory tissues in response to CS exposure; in contrast, e-vapor aerosol exposure had a milder impact. Oxidative stress caused by inhalation of CS is involved in the development of emphysema (Rangasamy et al. 2004, 2009) in part via oxidative stress is activation of the transcription factor nuclear factor-κB, which sustains pro-inflammatory cytokine transcription (Seagrave et al. 2004; Sharafkhaneh et al. 2008; Yang et al. 2006). Our CS results are aligned with those of other studies, which reported an effect of CS on lung inflammation and stress responses (Braber et al. 2010; Bracke et al. 2006; Thatcher et al. 2005). Other studies have reported that e-cigarette devices could generate carbonyl compounds, thus increasing pulmonary oxidative stress and inflammation (Cirillo et al. 2019; Ong et al. 2012). Carbonyl levels are device specific, depending on the puffing regimen or device setup (Margham et al. 2016; Thomson and Lewis 2015) the applied voltage and temperature of the heater coil (El-Hellani et al. 2018; Gillman et al. 2016; Kosmider et al. 2014; Sleiman et al. 2016), and the chemical composition of the liquids in the e-cigarette devices (Conklin et al. 2018). In CS, carbonyls are generated during pyrolysis, combustion and distillation of the tobacco smoke product, which reaches temperatures up to 900℃ (Baker et al. 2004). In the present study, the aerosols (carrier, base and test) were generated in a controlled manner using the CAG, which was set to 250–275℃ to match a representative temperature of the heated coil during puffing of e-cigarettes, leading to minimal or no detectable levels of carbonyls which could be one factor contributing to smaller impact on the respiratory system.

Our histopathological and morphometric analyses of the lungs revealed a decrease in the number of alveoli and alveolar surface density and an increase in the destructive index and mean chord length in response to 3R4F CS; these findings are characteristic of emphysematous lung changes (Sharafkhaneh et al. 2008). However, such emphysematous alterations were not observed in mice exposed to any of e-vapor aerosols. Exposure to 3R4F CS, but not exposure to e-vapor aerosols, induces a preeminent inflammatory response, which increases proteolytic activity and could contribute to the destruction of the extracellular matrix and development of emphysema (Foronjy et al. 2008; Sandhaus and Turino 2013). Our data are aligned with previous reports on the effect of CS on lung inflammation and stress responses (Braber et al. 2010; Bracke et al. 2006; Thatcher et al. 2005).

In a similar study, Madison et al. compared the pulmonary effects of e-vapor aerosol (with nicotine or carrier) to those of air and CS exposure in female mice (Madison et al. 2019). As in our study, analysis of BALF inflammatory cytokines, Mmp12 expression, cytometry analysis, and histopathological analysis of lung tissues showed no significant adverse effects of e-vapor aerosol exposure, despite these authors applying a different aerosol generation methodology. Madison et al., also concluded that e-vapor aerosol exposure did not cause lung inflammation and did not induce emphysematous changes. In the second part of this investigation, however, Madison et al. demonstrated that e-vapor aerosol exposure increased phospholipid accumulation in macrophages and altered Sftpd and Sfpta expression in the lungs. In contrast to the findings of Madison et al., our proteomics and lipidomics analyses were conducted on whole lung tissue and not on isolated macrophages and did not resolve such macrophage-specific changes. Our surfactant protein analysis results show, however, that only CS exposure significantly affected the expression of Sftpa1 and Sftpd (Fig. 5c), whereas no significant changes were observed in response to e-vapor aerosol exposure. Although both studies did analyze lipidomics, transcriptomics, and surfactant protein changes, the specificity of tissue (macrophages versus whole lungs used in our study) and dose of exposure used appear different, and this might have led to apparent disparate molecular observations. At the same time, it is important to note that the Madison et al. study included no CS reference in the quantitative comparison of molecular lipid metabolism alterations and provided no methodological details for example on urine collection and analysis, which makes it difficult to assess the biological relevance of the authors’ observations.

Our additional analysis of upper respiratory tract tissues demonstrated the absence of statistically significant adaptive changes in the nose following aerosol exposure. The only notable findings in the olfactory epithelium were that the eosinophilic globule scores at nose levels 3 and 4 were more pronounced following aerosol exposure (irrespective of the presence or absence of nicotine) than following 3R4F CS exposure. Although the long-term implication of localized changes is unknown, intraepithelial eosinophilic globules are regarded a common finding in nasal and respiratory tract tissues (Lewis et al. 1994) in inhalation studies and eosinophilic globules often coexist with other adaptive and/or degenerative changes such as metaplasia, hyperplasia, and atrophy of the nasal epithelia. They were proposed to be part of a continuum of changes in response to inhaled test substances (Aiso et al. 2005; Pauluhn 2012) and are potentially linked to cellular apoptosis (Papadimitriou et al. 2000).

The exact reason for this increased accumulation of intraepithelial globules in the olfactory epithelia in the aerosol-exposed groups is unclear, as it was not observed in previous rat inhalation studies involving PG-, VG-, nicotine- or flavor-containing aerosols (Ho 2018; Phillips et al. 2017; Werley et al. 2016). Neither of these findings (i.e., accumulation of intraepithelial globules or adaptive changes in respiratory and olfactory epithelia) was observed in response to heated-tobacco products (Phillips et al. 2019, 2016). While further investigation is desirable, the observed epithelial changes in the aerosol-exposed groups did not accompany degenerative characteristics. Consequently, the implication of these findings is considered minimal.

## Conclusions

Overall, this systems toxicology study investigated the impact of e-vapor aerosols generated using a laboratory capillary aerosol generator on the respiratory system in the *Apoe*^−/−^ mouse model. Our structural and functional findings demonstrate that, in contrast to CS exposure, e-vapor aerosol exposure preserved lung function and did not cause major alterations in the respiratory system, such as emphysematous and inflammatory changes. In comparison with sham exposure, aerosol exposures did not elicit adverse effects on most functional and histological endpoints; there was, however, a localized irritative effect (without degenerative characteristics) in the route of entry (nasal olfactory epithelium at levels 3 and 4). On the molecular level, aerosol exposure led to a significantly reduced dysregulation of the transcriptome, lipidome, and proteome relative to CS exposure. Altogether, our results show that e-vapor aerosols with or without flavor cause significantly fewer adverse effects associated with COPD in the respiratory tract than CS exposure.

## Supplementary Information

Supplementary material 1 (pdf 9164 KB)

## Data Availability

Datasets and additional data visualizations can be accessed at https://doi.org/org/10.26126/intervals.8lafdu.1. The data were deposited in the ArrayExpress database (https://www.ebi.ac.uk/arrayexpress/) under accession number (E-MTAB-8578).
