# Supplementary material for: A 6-month inhalation toxicology study in Apoe−/− mice demonstrates substantially lower effects of e-vapor aerosol compared with cigarette smoke in the respiratory tract
Source: Arch Toxicol. 2021 May 7;95(5):1805–29. doi: 10.1007/s00204-021-03020-4 (PMC8113187; doi:10.1007/s00204-021-03020-4)
Supplement: Supplementary file 1 — Supplementary material 1 (pdf 9164 KB) [file 204_2021_3020_MOESM1_ESM.pdf]

Online Resource 1

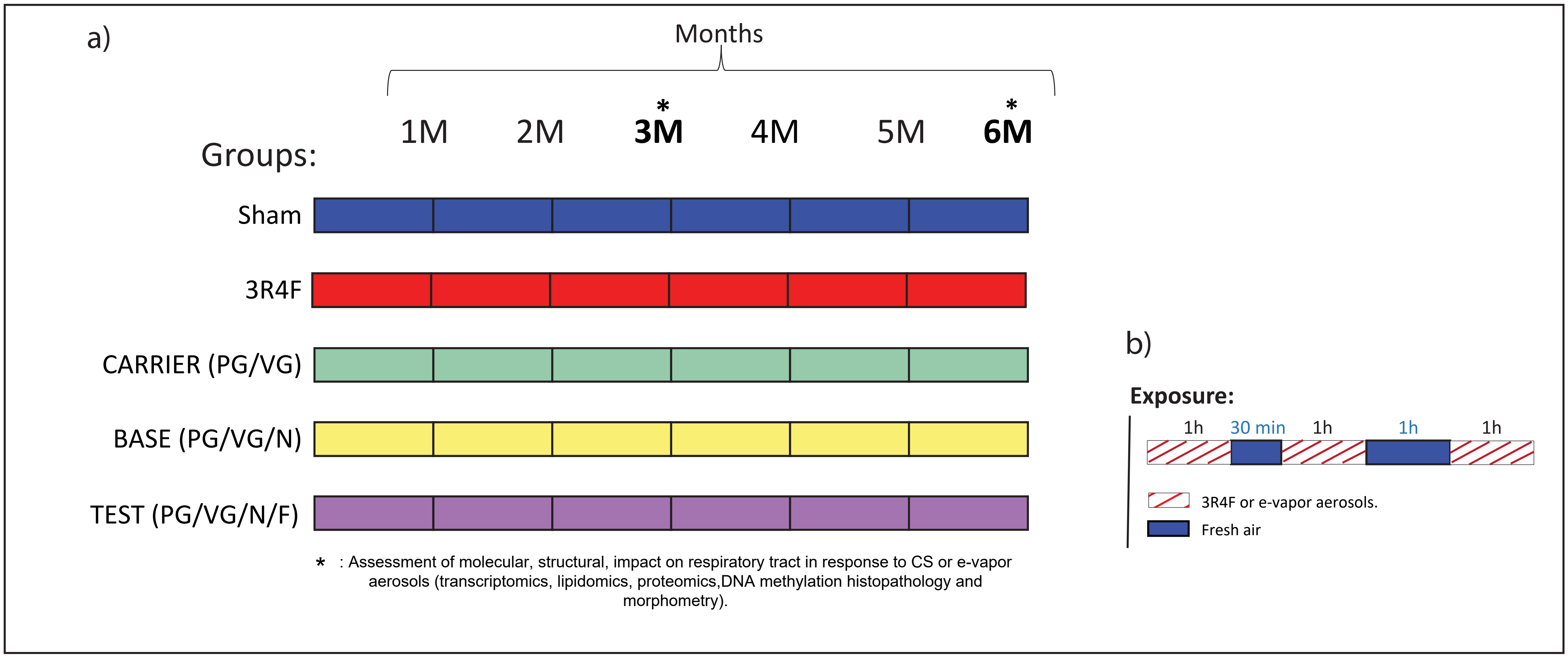

c) Allocations of mice to groups and endpoints

	BALF Analysis	HISTOPATHOLOGICAL Analysis	OMICS Analysis	MICRO CT	EXPOSED	TOTAL NUMBER OF MICE
Sham_3M	10	12	10	16		48
Sham_6M	10	12	10	16		48
Sham_R					7	7
3R4F_3M	10	12	10	16		48
3R4F_6M	10	12	10	16		48
3R4F_R					7	7
Carrier (PG/VG)_3M	10	12	10	16		48
Carrier (PG/VG)_6M	10	12	10	16		48
Carrier (PG/VG)_R					7	7
Base (PG/VG/N)_3M	10	12	10	16		48
Base (PG/VG/N)_6M	10	12	10	16		48
Base (PG/VG/N)_R					7	7
Test (PG/VG/N/F)_3M	10	12	10	16		48
Test (PG/VG/N/F)_6M	10	12	10	16		48
Test (PG/VG/N/F)_R					7	7
TOTAL	100	120	100	160	35	515

Online Resource 1: Study design, groups, and exposure. (a) Timeline of the study, showing the experimental groups. 3R4F is the standard reference cigarette; carrier, base, and test represent e-vapor aerosols from the test formulations. “Carrier” contains propylene glycol and vegetable glycerol; “base” contains propylene glycol, vegetable glycerol, and nicotine; “test” contains propylene glycol, vegetable glycerol, nicotine, and flavor. (b) Whole-body exposure protocol. The red dashed square represents the exposure time. The blue square represents the exposure break. (c) Allocation of mice to groups and endpoints (for details, see text in Materials and methods).

Online Resource 2

Study design

This work is part of a comprehensive inhalation study that also included assessment of systemic and cardiorespiratory effects in the same mouse model (Szostak et al. 2020). The experimental animals were approximately 12–14 weeks old at the start of exposure. The mice were randomized into five exposure groups: Sham (exposed to fresh air), 3R4F reference cigarette ("3R4F"), PG/VG ("carrier"), PG/VG/nicotine ("base"), and PG/VG/nicotine/flavoring ("test"). Exposed reserve mice (n = 35) were used to replace moribund animals or premature deaths at any point during the study **(Online Resource 1c).** The aerosol for the base group contained PG, VG, and 4% nicotine, while that for the test group contained PG, VG, 4% nicotine, and flavor mix. The animals were exposed to 3R4F CS or E-vapor aerosols for 3 h per day, 5 days per week, in whole-body exposure chambers. The base and test group exposures were configured to deliver a nicotine concentration of 35 µg/L (equivalent to the nicotine level in 560 µg/L total particulate matter (TPM) from 3R4F CS). The exposure time and fresh-air-break regimens were consistent across exposure groups. The maximum exposure duration was 6 months, and interim dissection points were scheduled after 3 and 6 months (**Online Resource 1a**). Exposed reserve mice (n = 7 per exposure condition) were used to replace moribund animals or premature deaths at any point during the study (**Online Resource 1c**). Aerosols were generated from liquid formulations as indicated in **(Online Resource 1c).** At each dissection point, the animals were allocated to the following endpoints: bronchoalveolar lavage fluid (BALF) analysis, including analysis of free lung cells and multianalyte (cytokines/chemokines) profiling; histopathological evaluation of respiratory tract organs and morphometry of lungs; lung function analysis; and molecular analysis (transcriptomics, proteomics, lipidomics, and DNA methylation). Respiratory tissues were collected from various groups for transcriptomics, lipidomics, proteomics, genomics, histopathological, immunohistochemical, analysis **(Online Resource 1c).**

Test atmosphere generation

In cigarettes smoke, carbonyls are generated during pyrolysis and distillation of the tobacco product, which reaches temperatures close to 600°C (Baker et al. 2004). The CAG system controls the heating process at 250°C, leading to markedly reduced concentrations of carbonyls in the test atmosphere relative to CS. The CAG was developed by Philip Morris, Inc.(Gupta et al. 2003; Howell and Sweeney 1998); it was previously shown to deliver aerosol in a consistent manner and at similar particle size distribution and concentrations as a prototype ciga-like e-cigarette (Werley et al. 2016). The CAG aerosol in this study was generated from an e-liquid composed of typical constituents

at high purity, under defined heating and flow conditions. The particle size distribution obtained (mass median aerodynamic diameter [MMAD], 0.81–1.01 µm; geometric standard deviation [GSD], 1.27–1.43) was reproducible, and we consider them representative for commercial electronic nicotine delivery systems (ENDS): Oldham and colleagues recently reported MMADs ranging from 0.9 to 1.2 µm and GSDs ranging from 1.7 to 2.2 for aerosols generated with 20 different commercial ENDS (Oldham et al. 2018).

The CAG was fitted with a diffuser and compressed air to prevent aerosol backflow. Condensation aerosol was created when output vapor from the generator was mixed with dilution air at ambient temperature. The aerosol was further diluted with filtered air to achieve the target concentrations in the test atmosphere and delivered via glass tubing to the whole-body exposure chambers. The aerosols generated from carrier, base, and test formulations are hereafter termed e-vapor aerosols.

The atmosphere in the aerosol exposure chambers was monitored for flow rate, temperature, relative humidity, aerosol mass concentration (TPM), and particle size as well as the concentrations of carbon monoxide (CO), formaldehyde, acetaldehyde, propionaldehyde, crotonaldehyde and acrolein. The detailed description of the procedures were described in previously published work (Phillips et al. 2016; Phillips et al. 2015). Longitudinal monitoring of nicotine and TPM concentrations is shown in **Online Resource 7a and 7b.**

Animals and inhalation exposure

The animals' health status was verified by using the health-check certificate provided by the breeder. Additional health checks were performed at month 2 (blood from health-check animals was sent for serological analysis) and at the end of study (live animals in the health-check group were sent for health checks). The mice were maintained and exposed under specific hygienic conditions, with filtered conditioned fresh air at 22±3°C and 30–70% humidity. Additional details on animal housing, randomization, and acclimatization have been published previously (Boue et al. 2012; Lietz et al. 2013; Phillips et al. 2016).

BALF collection

Briefly, BALF collection consisted of five lavage cycles. The first cycle was performed by using phosphate-buffered saline (Thermo Fisher Scientific, Waltham, MA, USA). In the remaining four cycles, BALF was collected by using phosphate-buffered saline with 0.325% bovine serum albumin (Millipore Sigma, St. Louis, MO, USA). The BALF samples were centrifuged to obtain the cell-free supernatants and cell pellets. Cell pellets pooled from the five collection cycles were processed for flow cytometric analysis of free lung cells, and BALF supernatants (from the first

collection cycle) were stored as aliquots at ≤−70°C until analysis of inflammatory mediators and gelatinolytic matrix metalloproteinase (MMP) activity.

Analysis of free lung cells in BALF

Following determination of cell count, free lung cells were analyzed by flow cytometry after staining with cell type-specific antibodies conjugated to fluorochromes. Both absolute and relative counts of macrophages, neutrophils, lymphocytes, and their subtypes were reported. Relative counts can be assessed at https://www.intervals.science/studies#/search/.

Analysis of inflammatory mediators and MMP activity in BALF

Inflammatory mediators in the BALF supernatant were analyzed on a FLEXMAP 3D® platform equipped with the xPONENT® software version 4.2 (Luminex Corp., Austin, TX, USA) by using the Millipore multiplexed bead array (Millipore Sigma; reference: MCYTOMAG-70K-PMX-25 and MCYTOMAG-77K). Gelatin MMP proteolytic activity was determined by using a commercially available kit (EnzChek® Gelatinase/Collagenase Assay Kit; Thermo Fisher Scientific)., location?

Histopathological analysis and morphometry

Peer review was conducted contemporaneously by an independent pathologist (Laboratory of Pharmacology and Toxicology GmbH, Hamburg, Germany) on the evaluation of the RNE, larynx and lungs in the sham, 3R4F, PG/VG/N, and PG/VG/N/F groups from the month 6 scheduled dissection. The results from a total of 10 mice per group were peer-reviewed. An agreement on the final diagnosis of the histopathology findings was reached at the end of the peer review.

Histopathological analysis and morphometry

The left lung lobe was serially sectioned (4-µm thickness) into step sections 300 µm apart for overall assessment of the entire lung lobe. Representative paraffin sections were also obtained from the nose (four predefined levels at posterior to the upper incisor teeth, posterior to the incisive papilla, at the second *ruga palatina*, and between the first and second molar teeth), larynx (at the base of the epiglottis and arytenoid projections), and trachea (transverse section at the thyroid gland and longitudinal section at the bifurcation). The sections were stained with hematoxylin and eosin

and the Alcian blue–periodic acid–Schiff reagent for the left lung and nose (for polysaccharides and glycoproteins, including mucus) and resorcin–fuchsin for the left lung (elastic fibers).

Digitalized (ScanScope, Aperio Technologies, Vista, CA, USA) serial sections of the lungs were also evaluated by morphometry. Morphometry was performed by using a design-based stereological approach to obtain quantitative data (newCAST™, Visiopharm, Hoersholm, Denmark) addressing the characteristics of alveolar emphysema (i.e., tissue destruction and hyperinflation) by various endpoints. Quantitative lung morphometry was performed by a board-certified veterinary pathologist in a blinded fashion (Histovia GmbH).

Lung, respiratory nasal epithelium, and trachea transcriptomics analyses

The acceptance criterion for downstream processing of the RNA samples was an RNA integrity number >6. For mRNA analysis, 100 ng of total RNA was reverse-transcribed to cDNA by using the Affymetrix® HT 3′ IVT PLUS kit (Thermo Fisher Scientific). cDNA was labeled and amplified to cRNA, which was then fragmented and hybridized to a GeneChip Mouse Genome 430 2.0 Array (Thermo Fisher Scientific). The arrays were washed and stained on an FS450 DX GeneChip® Fluidics Station (Thermo Fisher Scientific) by using the FS450_0001 protocol, and then scanned by using a 3000 7G GeneChip® scanner (Thermo Fisher Scientific). Raw data files were processed with custom computable document format files from a brain array (mouse4302mmentrezg v16.0) and normalized by frozen robust microarray analysis (McCall et al. 2010). Following quality control procedures (pseudoimages, normalized unscaled standard error plot, relative log expression plots, and median absolute value relative log expression), raw p values were generated for the treatment versus control (sham) comparisons by using the *limma* package in R and adjusted by Benjamini–Hochberg false discovery rate (FDR) multiple test correction (Benjamini et al. 2001; Gautier et al. 2004; Smyth 2004). FDR-adjusted p values <0.05 were considered significant.

Lung lipidomics analysis

Lipidomics profiles of lungs (N = 10) were generated using a high-resolution tandem mass spectrometry (MS/MS) shotgun lipidomics protocol. All samples were analyzed in random order and split into batches of up to 32 samples each. All batches included blank and QC samples (mouse pooled EDTA plasma, BioIVT, Westbury, NY, USA) to monitor the performance of the quantification workflow. Frozen lung tissue slices were homogenized in 150 mM ammonium bicarbonate buffer using a Branson W-450D Digital Sonifier (Branson, Danbury, CT, USA). The total protein content of tissue lysates was determined by the Bradford assay (Quick Start™, Bio-Rad, Hercules, CA, United States). Aliquots of tissue lysates were spiked with an internal deuterated standard mixture (Mouse Splash

Lipidomics(Avanti Polar Lipids, Alabaster, AL, USA) and extracted by the butanol-methanol (BUME) method (Lofgren et al. 2012). High-resolution direct infusion MS-MS/MS was performed both in positive and negative ionization modes on a Q Exactive™ Plus Orbitrap (Thermo Scientific, city, Germany) equipped with a Triversa NanoMate robotic interface (Advion, Ithaca, NY, USA). NanoMate parameters were set to 1.25-psi gas pressure and 1.1-kV voltage over a 5-min delivery time. The MS source settings were fixed at a column temperature of 250°C and S-lens radio frequency (RF) level of 65.0. The MS method for the positive ionization mode involved 1 min of full scan covering the m/z range from 550 to 1000 at 140,000 x resolution, with 10E6 automated gain control, a maximum injection time of 50 ms, and a lock mass of 680.48022. The 1- to 5-min DIA MS/MS acquisition was triggered with first mass fixed at 250 m/z, resolution of 17,500 x, automated gain control of 1E5, and a maximum injection time of 64 ms at 20 Normalized Collision Energy™ (NCE). An isolation window of 1 m/z was set, with the inclusion mass list starting from 550 to 1000. The MS method for the negative ionization mode involved 1 min of full scan covering the m/z range from 400 to 940 at 140,000 x resolution, with 1E6 automated gain control, a maximum injection time of 50 ms, and a lock mass of 529.46262. Then for 1 to 5 min, DIA MS/MS acquisition was triggered with first mass fixed at 150 m/z, resolution of 17,500 x, automated gain control of 1E5, and a maximum injection time of 64 ms at 35 NCE, with the inclusion mass list starting from 400 to 940 with a mass step of 1 Da.

Raw files were converted to mzML using PeakByPeak software (SpektroSwiss, Lausanne, Switzerland), which provides automated noise subtraction. The converted files were processed with LipidXplorer v.1.2.7.(Herzog et al. 2012). Lipid identification was performed by MS/MS, and quantification of identified species was performed using the MS level. Different lipid species of PC, PE, PS, phosphatidylinositol (PI), phosphatidic acid (PA), phosphatidylglycerol (PG), sphingomyelin (SM), diacylglycerol (DAG), and triacylglycerol (TAG) were listed with the sum of fatty acyl groups (e.g., PC 32:0). LPC and LPE refer to lyso-PC and lyso-PE, respectively, and sterol/cholesteryl esters are abbreviated SE. Ether-linked phospholipids are shown as PCO (alkyl) and PEO (alkyl).

The final lipid concentration was normalized per amount of total protein. A linear model was fitted for each exposure condition and the corresponding air-exposed group, and *p* values from a *t*-test were calculated for $\log_2$-transformed data (Gentleman et al. 2004). The Benjamini–Hochberg FDR method was used to correct for multiple testing effects. Lipids with an adjusted *p* value <0.05 were considered differentially abundant

Lung proteomics analysis

Proteome alterations in the lungs (N = 10) were assessed by isobaric tag-based quantification using the iTRAQ® approach. Samples were processed and analyzed in randomized order. Frozen lung tissue slices were homogenized

using a bead-assisted procedure in a TissueLyser II (Qiagen, Hilden, Germany) in Tissue Lysis Buffer (BioRad Laboratories, Hercules, CA, USA) prior to acetone precipitation (in random order). Protein precipitates were resuspended in 0.5 M triethylammonium bicarbonate (Sigma-Aldrich, St. Louis, MO, USA), 1 M urea (Sigma-Aldrich), and 0.1% sodium dodecyl sulfate (Sigma-Aldrich). Protein concentrations were determined by the Bradford assay (Quick Start™, Bio-Rad, Hercules, CA, United States). Next, 50-µg aliquots of the suspension were processed using the iTRAQ® 8-plex labeling procedure in accordance with the manufacturer's instructions (AB Sciex, Framingham, MA, USA). A trypsin–Lys C mix (Promega, Madison, WI, USA) was added to the samples at a 1:10 ratio (w/w), followed by overnight digestion at 37°C. Trypsin-digested samples were labeled with reporter-ion tags for different exposure groups.

Separately for each analysis time point, iTRAQ® analysis sets with randomized set and reporter ion channel assignments were defined: Each 8-plex labeling replicate set included one sample of each exposure group and one pooled reference mix combining all samples. All labeled samples that belonged to one iTRAQ® replicate set were pooled and dried in a SpeedVac concentrator (RVC 2–25 CD Plus; Martin Christ, Osterode am Harz, Germany). The samples were desalted using 0.5-mL Detergent Removal Columns (Pierce, Rockford, IL, USA) and with Sep-Pak C18 1-cc reverse-phase columns (Waters, Milford, MA, USA) in accordance with the manufacturers' protocols. The samples were dried in a SpeedVac evaporator, resuspended in nanoLC buffer A (5% acetonitrile, 0.2% formic acid; Sigma-Aldrich), and analyzed in random order using an Easy nanoLC 1000 instrument connected online to a Q Exactive™ mass analyzer (Thermo Fisher Scientific). Peptides were separated on a 50-cm Acclaim™ PepMap™ 100 C18 LC column (2-µm particle size; Thermo Fisher Scientific) at a flow rate of 200 nL/min, with a 200-min gradient from nanoLC buffer A to 40% acetonitrile with 0.2% formic acid. Each sample was injected twice, with two different analytical methods on the same column (one fast and one sensitive method), as previously described (Kelstrup et al. 2014). The outputs of both MS runs were combined as merged mass-lists and interrogated against the mouse reference proteome set (UniProt, version July 2014, canonical isoforms only) using Proteome Discoverer version 1.4 (Thermo Fisher Scientific). SequestHT (implemented in Proteome Discoverer) was used as the search tool, and iTRAQ® reporter-ion intensities were determined from Proteome Discoverer. The Percolator node of Proteome Discoverer was used to estimate peptide-level FDR-adjusted p values (q values).

iTRAQ® peptide-level quantification data were exported and further processed in the R statistical environment (R Development Core Team 2007). Quantification data were filtered for q values <0.01 and "unique" quantification results as defined by Proteome Discoverer. A global variance-stabilizing normalization was performed with the corresponding Bioconductor package in R (Huber et al. 2002; Hultin-Rosenberg et al. 2013). Each iTRAQ® reporter-ion set was

normalized to its median, and protein expression values were calculated as the medians of these normalized peptide-level quantification values (Herbrich et al. 2013).

For the statistical analysis, a linear model was fitted for each exposure condition and the respective Sham group, *p* values were calculated from moderated *t*-statistics with the empirical Bayes approach (Gentleman et al. 2004), and proteins with a Benjamini–Hochberg FDR-adjusted *p* value <0.05 were considered differentially expressed.

Lung whole-genome methylation analysis

Data processing was performed as previously described (Phillips et al. 2019). Briefly, sequencing reads were aligned to the mouse genome (mm10), and methylation was quantified at CpG sites by using the QuasR package in Bioconductor. DNA methylation changes were computed at *cis*-regulatory elements that are promoters (transcription start sites ±0.5 kb) and candidate enhancers, identified as low-methylated regions (Burger et al. 2013). The significance of differential methylation was assessed by using the FDR adjusted *p* value from the beta/binomial model. Read cutoff was set to 15.

Statistical analysis for apical endpoints

For continuous variables, if the data of the two groups being compared did not exhibit strong deviation from the normal distribution (as assessed by a Shapiro–Wilk test at a 5% level on the standardized residuals of both groups), a two-sample *t*-test accounting for variance heterogeneity was performed. Otherwise, an exact Mann–Whitney–Wilcoxon two-sample test was used (Monte Carlo estimates of the exact *p* values were used). The Cochran–Mantel–Haenszel test was used for score variables (histopathology), and Fisher's exact test was used for incidence variables. All analyses were performed with SAS version 9.2 (SAS Institute Inc., Cary, NC, USA).

Rationale for the choice of the model

Chronic obstructive pulmonary disease (COPD) is a complex disease that includes both emphysema and airway remodeling, and female hormones may influence these pathologic changes. COPD was historically considered to be a disease that mainly affected senior men, reflecting the high prevalence of smoking among men (Barnes 2016). However, in many developed countries, COPD becomes more prevalent in women than men (Gan et al. 2006; Mannino and Buist 2007) and it was suggested that women are more susceptible to developing COPD in response to cigarette smoke exposure than men. Indeed, epidemiological studies showed that females suffer more severe COPD with early-onset disease (<60 yr) and greater susceptibility to COPD with lower tobacco exposure, compared to men (Aryal et al. 2013; Dransfield et al. 2006; Sorheim et al. 2010).

A similar trend with an increase of COPD susceptibility and severity was observed in the mice. Van Winkle and colleagues have demonstrated that female mice showed more significant proximal airway injury than male mice after acute injection of naphthalene, an important constituent of side-stream cigarette smoke (Van Winkle et al. 2002). Sex differences analysis in scientific literature tends to highlight that female mice have a higher susceptibility to emphysematous changes than male. For example, female A/J mice developed emphysema sooner than male A/J mice: after 10 weeks and 16 weeks of cigarette smoke exposure, respectively (March et al. 2006). In the C57BL/6 mice model, airway remodeling in female mice following chronic smoke exposure is associated with increased tissue resistance in the peripheral airways (Tam et al. 2016). Also the recent inhalation study by Madison et al. (Madison et al. 2019) was conducted exclusively on female mice. Whereas some recent publications encourage scientists to use biological sex as experimental variable to account for the potential effect of sex hormones (Blenck et al. 2016; Dworatzek and Mahmoodzadeh 2017; Mouat et al. 2018; Regitz-Zagrosek and Kararigas 2017; Ventura-Clapier et al. 2017), we chose, in part considering the "3Rs" (Reduce, Refine, Replace), to study only one susceptible sex in order to reduce the number of animals while performing an in depth investigation of the cardiorespiratory system at molecular, structural, and functional levels. Although a variety of mouse models are used for evaluating atherosclerosis (ApoE-/-, LDLR-/-, or double knockouts), it should be recognized that al these models may not present the exact characteristics of human disease and the atherosclerotic lesions are developed in different vessel types and locations (Badimon 2001), suggesting some mechanistic differences.

.

References:

Aryal S, Diaz-Guzman E, Mannino DM (2013) COPD and gender differences: an update Translational Research 162:208-218 doi:https://doi.org/10.1016/j.trsl.2013.04.003

Badimon L (2001) Atherosclerosis and thrombosis: lessons from animal models Thromb Haemost 86:356-365

Baker RR, Massey ED, Smith G (2004) An overview of the effects of tobacco ingredients on smoke chemistry and toxicity Food and chemical toxicology : an international journal published for the British Industrial Biological Research Association 42 Suppl:S53-83 doi:10.1016/j.fct.2004.01.001

Barnes PJ (2016) Sex Differences in Chronic Obstructive Pulmonary Disease Mechanisms Am J Respir Crit Care Med 193:813-814 doi:10.1164/rccm.201512-2379ED

Benjamini Y, Drai D, Elmer G, Kafkafi N, Golani I (2001) Controlling the false discovery rate in behavior genetics research Behavioural brain research 125:279-284

Blenck CL, Harvey PA, Reckelhoff JF, Leinwand LA (2016) The Importance of Biological Sex and Estrogen in Rodent Models of Cardiovascular Health and Disease Circ Res 118:1294-1312 doi:10.1161/CIRCRESAHA.116.307509

Boue S et al. (2012) Modulation of atherogenic lipidome by cigarette smoke in apolipoprotein E-deficient mice Atherosclerosis 225:328-334 doi:10.1016/j.atherosclerosis.2012.09.032

Burger L, Gaidatzis D, Schubeler D, Stadler MB (2013) Identification of active regulatory regions from DNA methylation data Nucleic Acids Res 41:e155 doi:10.1093/nar/gkt599

Dransfield MT, Davis JJ, Gerald LB, Bailey WC (2006) Racial and gender differences in susceptibility to tobacco smoke among patients with chronic obstructive pulmonary disease Respir Med 100:1110-1116 doi:10.1016/j.rmed.2005.09.019

Dworatzek E, Mahmoodzadeh S (2017) Targeted basic research to highlight the role of estrogen and estrogen receptors in the cardiovascular system Pharmacol Res 119:27-35 doi:10.1016/j.phrs.2017.01.019

Gan WQ, Man SF, Postma DS, Camp P, Sin DD (2006) Female smokers beyond the perimenopausal period are at increased risk of chronic obstructive pulmonary disease: a systematic review and meta-analysis Respir Res 7:52 doi:10.1186/1465-9921-7-52

Gautier L, Cope L, Bolstad BM, Irizarry RA (2004) affy--analysis of Affymetrix GeneChip data at the probe level Bioinformatics 20:307-315 doi:10.1093/bioinformatics/btg405

Gentleman RC et al. (2004) Bioconductor: open software development for computational biology and bioinformatics Genome biology 5:R80

Gupta R, Hindle M, Byron PR, Cox KA, McRae DD (2003) Investigation of a novel condensation aerosol generator: solute and solvent effects Aerosol Science & Technology 37:672-681

Herbrich SM et al. (2013) Statistical Inference from Multiple iTRAQ Experiments without Using Common Reference Standards Journal of proteome research 12:594-604

Herzog R, Schuhmann K, Schwudke D, Sampaio JL, Bornstein SR, Schroeder M, Shevchenko A (2012) LipidXplorer: a software for consensual cross-platform lipidomics PloS one 7:e29851

Howell TM, Sweeney WR (1998) Aerosol and a method and apparatus for generating an aerosol

Huber W, Von Heydebreck A, Sültmann H, Poustka A, Vingron M (2002) Variance stabilization applied to microarray data calibration and to the quantification of differential expression Bioinformatics 18:S96-S104

Hultin-Rosenberg L, Forshed J, Branca RM, Lehtio J, Johansson HJ (2013) Defining, comparing, and improving iTRAQ quantification in mass spectrometry proteomics data Molecular & cellular proteomics : MCP 12:2021-2031 doi:10.1074/mcp.M112.021592

Kelstrup CD, Jersie-Christensen RR, Batth TS, Arrey TN, Kuehn A, Kellmann M, Olsen JV (2014) Rapid and deep proteomes by faster sequencing on a benchtop quadrupole ultra-high-field Orbitrap mass spectrometer Journal of proteome research 13:6187-6195

Lietz M et al. (2013) Cigarette-smoke-induced atherogenic lipid profiles in plasma and vascular tissue of apolipoprotein E-deficient mice are attenuated by smoking cessation Atherosclerosis 229:86-93 doi:10.1016/j.atherosclerosis.2013.03.036

Lofgren L, Stahlman M, Forsberg G-B, Saarinen S, Nilsson R, Hansson GI (2012) The BUME method: a novel automated chloroform-free 96-well total lipid extraction method for blood plasma Journal of lipid research:jlr. D023036

Madison MC et al. (2019) Electronic cigarettes disrupt lung lipid homeostasis and innate immunity independent of nicotine J Clin Invest doi:10.1172/JCI128531

Mannino DM, Buist AS (2007) Global burden of COPD: risk factors, prevalence, and future trends Lancet 370:765-773 doi:10.1016/S0140-6736(07)61380-4

March TH et al. (2006) Modulators of cigarette smoke-induced pulmonary emphysema in A/J mice Toxicol Sci 92:545-559 doi:10.1093/toxsci/kfl016

McCall MN, Bolstad BM, Irizarry RA (2010) Frozen robust multiarray analysis (fRMA) Biostatistics 11:242-253 doi:10.1093/biostatistics/kxp059

Mouat MA, Coleman JLJ, Smith NJ (2018) GPCRs in context: sexual dimorphism in the cardiovascular system Br J Pharmacol 175:4047-4059 doi:10.1111/bph.14160

Oldham MJ, Zhang J, Rusyniak MJ, Kane DB, Gardner WP (2018) Particle size distribution of selected electronic nicotine delivery system products Food and chemical toxicology : an international journal published for the British Industrial Biological Research Association 113:236-240 doi:10.1016/j.fct.2018.01.045

Phillips B et al. (2019) A six-month systems toxicology inhalation/cessation study in ApoE(-/-) mice to investigate cardiovascular and respiratory exposure effects of modified risk tobacco products, CHTP 1.2 and THS 2.2, compared with conventional cigarettes Food and chemical toxicology : an international journal published for the British Industrial Biological Research Association 126:113-141 doi:10.1016/j.fct.2019.02.008

Phillips B et al. (2016) An 8-Month Systems Toxicology Inhalation/Cessation Study in Apoe-/- Mice to Investigate Cardiovascular and Respiratory Exposure Effects of a Candidate Modified Risk Tobacco Product, THS 2.2, Compared With Conventional Cigarettes Toxicol Sci 149:411-432 doi:10.1093/toxsci/kfv243

Phillips B et al. (2015) A 7-month cigarette smoke inhalation study in C57BL/6 mice demonstrates reduced lung inflammation and emphysema following smoking cessation or aerosol exposure from a prototypic modified risk tobacco product Food and chemical toxicology : an international journal published for the British Industrial Biological Research Association 80:328-345 doi:10.1016/j.fct.2015.03.009

R Development Core Team (2007) R: A Language and Environment for Statistical Computing.

Regitz-Zagrosek V, Kararigas G (2017) Mechanistic Pathways of Sex Differences in Cardiovascular Disease Physiol Rev 97:1-37 doi:10.1152/physrev.00021.2015

Smyth GK (2004) Linear models and empirical bayes methods for assessing differential expression in microarray experiments Statistical applications in genetics and molecular biology 3:Article3 doi:10.2202/1544-6115.1027

Sorheim IC, Johannessen A, Gulsvik A, Bakke PS, Silverman EK, DeMeo DL (2010) Gender differences in COPD: are women more susceptible to smoking effects than men? Thorax 65:480-485 doi:10.1136/thx.2009.122002

Szostak J et al. (2020) A 6-month systems toxicology inhalation study in ApoE(-/-) mice demonstrates reduced cardiovascular effects of E-vapor aerosols compared with cigarette smoke Am J Physiol Heart Circ Physiol 318:H604-H631 doi:10.1152/ajpheart.00613.2019

Tam A, Bates JH, Churg A, Wright JL, Man SF, Sin DD (2016) Sex-Related Differences in Pulmonary Function following 6 Months of Cigarette Exposure: Implications for Sexual Dimorphism in Mild COPD PLoS One 11:e0164835 doi:10.1371/journal.pone.0164835

Van Winkle LS, Gunderson AD, Shimizu JA, Baker GL, Brown CD (2002) Gender differences in naphthalene metabolism and naphthalene-induced acute lung injury Am J Physiol Lung Cell Mol Physiol 282:L1122-1134 doi:10.1152/ajplung.00309.2001

Ventura-Clapier R et al. (2017) Sex in basic research: concepts in the cardiovascular field Cardiovasc Res 113:711-724 doi:10.1093/cvr/cvx066

Werley MS, Miller IV JH, Kane DB, Tucker CS, McKinney Jr WJ, Oldham MJ (2016) Prototype e-cigarette and the capillary aerosol generator (CAG) comparison and qualification for use in subchronic inhalation exposure testing Aerosol Science and Technology:1-10

Online Resource 3

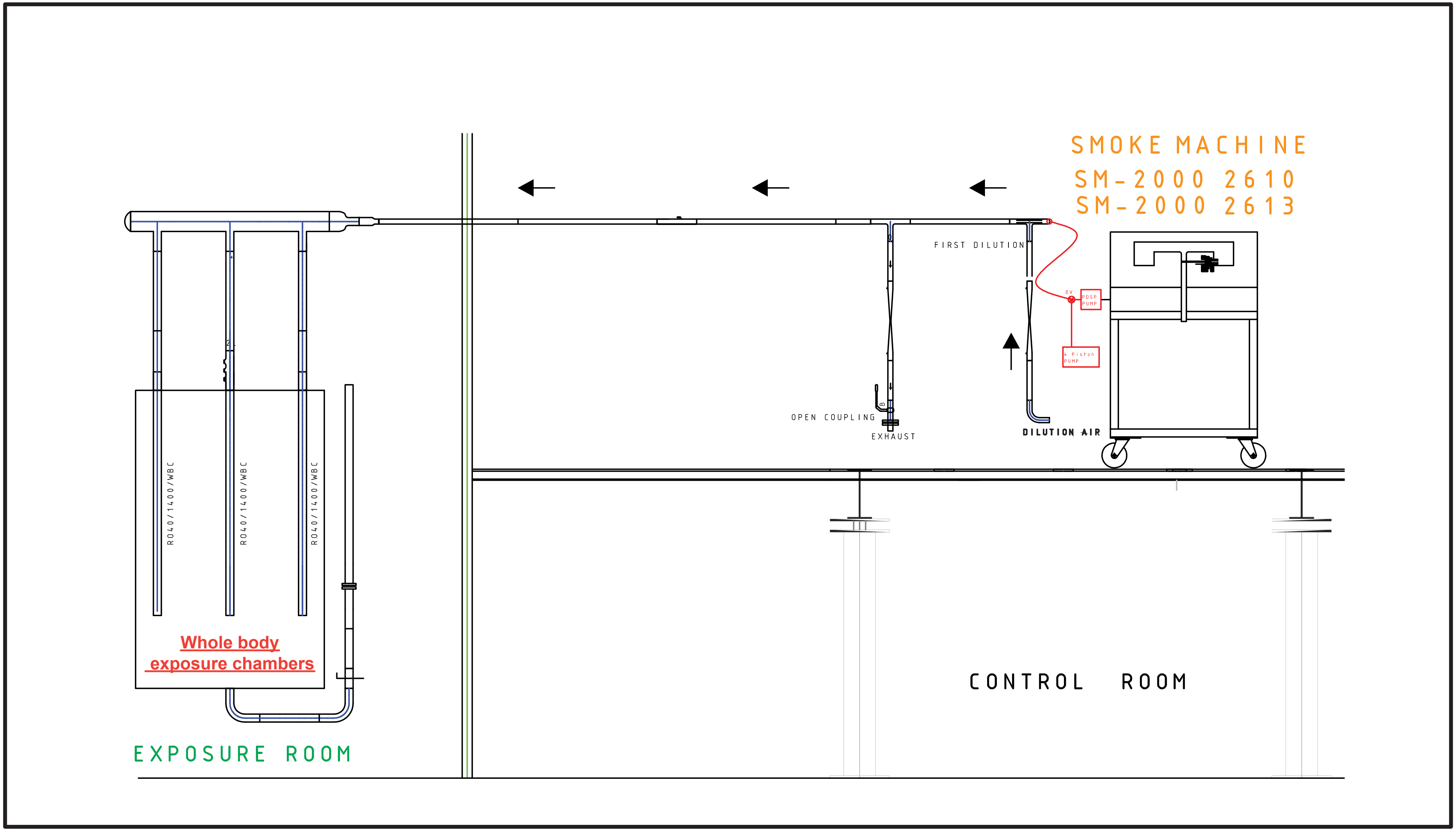

Online Resource 3: Schematic diagram of the generation and delivery of 3R4F cigarette smoke in the whole-body exposure chamber. Smoking machine and chamber layout for the sham and CS-3R4F exposure groups.

Online Resource 4

Mass composition of the tested liquids.

Component	CARRIER (PG/VG) (g/1000g)	BASE (PG/VG/N) (g/1000g)	TEST (PG/VG/N/F) (g/1000g)
Propylene glycol (PG)	255.00	240.00	238.91
Vegetable Glycerin (VG)	595.00	560.01	559.90
Water	150.00	150.00	150.00
Nicotine	0.00	40.00	40.00
Benzoic acid	0.00	3.33	3.33
Lactic acid	0.00	3.33	3.33
Acetic acid	0.00	3.33	3.33
Flavor blend	0.00	0.00	1.20
**Sum**	**1000.00**	**1000.00**	**1000.00**

Online Resource 5

Blended flavor mix in the test article formulation

3-METHYLPENTANOIC ACID
ACETYLPYRAZINE
METHYLBUTYRIC ACID
ISOVALERIC ACID
GUAIACOL
MEGASTIGMATRIENONE
ISOBUTYRALDEHYDE
4-Propylphenol
BENZENEMETHANOL
METHYL BUTANAL
METHOXY-4-METHYLPHENOL
KETOISOPHORONE
ETHYL-3-METHYLPYRAZINE
ETHYL-2-METHOXY-PHENOL
EUGENOL
METHYL-PENTANOIC ACID
DIMETHYL-1,2-CYCLOPENTADIONE
5-Methyl-6,7-dihydro-5H-cyclopenta[b]pyrazine
VANILLIN
2-ETHYL-3,(5 OR 6)-DIMETHYLPYRAZINE
PHENETHYL ALCOHOL
2,3,6-trimethylphenol
PENTEN-3-ONE
1-(2,6,6-TRIMETHYLCYCLOHEXA-1,3-DIEN-1-YL)-2-BUTEN-1-ONE
PHENYLACETIC ACID
DIMETHYL-3-HYDROXY-2,5-DIHYDROFURAN-2-ONE
BUTYRIC ACID
METHYLBUTYRALDEHYDE
DIETHYL-5-METHYL-PYRAZINE
DIETHYLPYRAZINE
2-methoxy-3-(1-methylpropyl)pyrazine
ACETYLPYRIDINE

Nicotine (data shown on Online Resource 4)

Online Resource 6

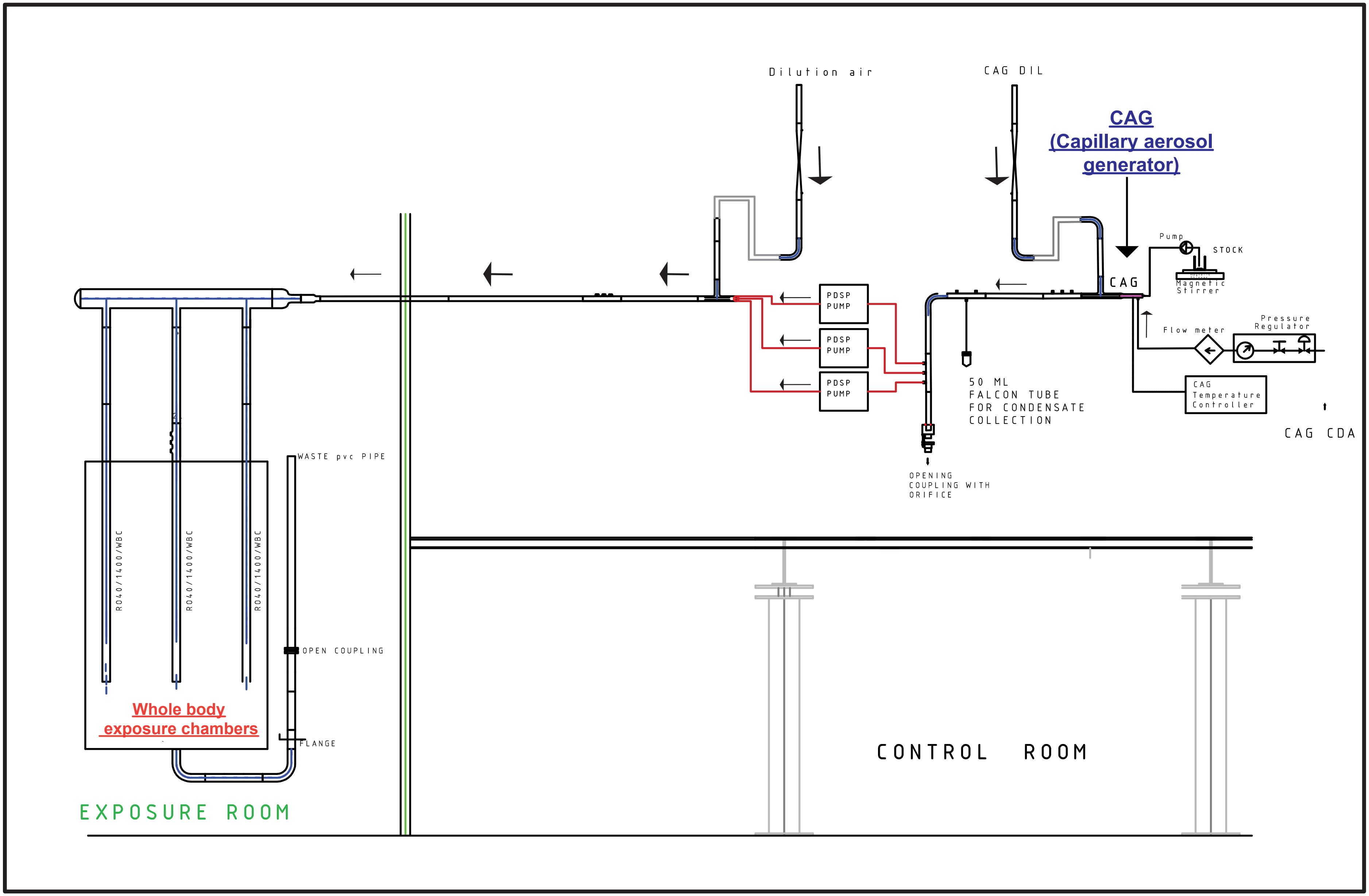

Online Resource 6: Schematic diagram of the generation and delivery of aerosols in the whole-body exposure chamber. Capillary aerosol generator and chamber layout for the carrier (propylene glycol and vegetable glycerol), base (propylene glycol, vegetable glycerol, and nicotine), and test (propylene glycol, vegetable glycerol, nicotine, and flavor) groups.

Online Resource 7

Exposure regimen (nicotine level) during the 6 months expsure.

(a)

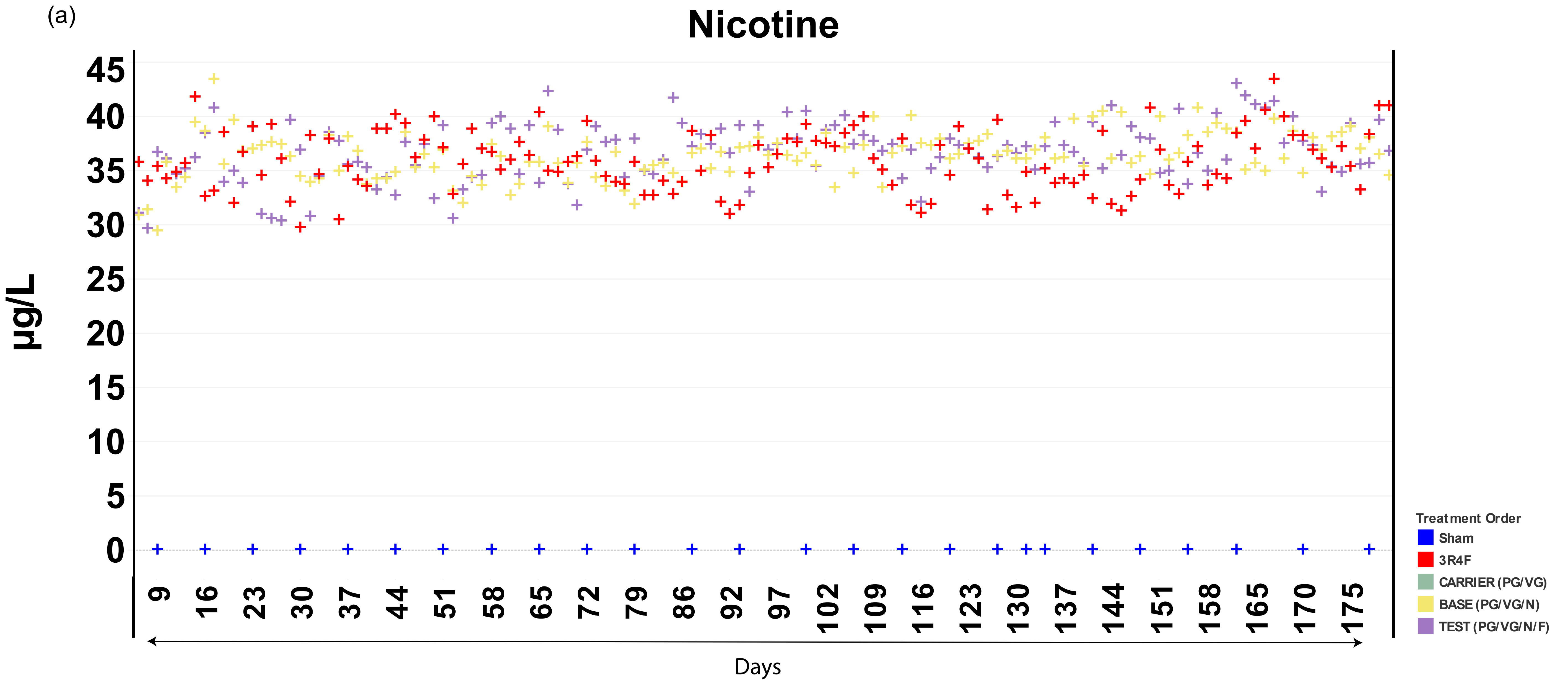

Exposure regimen (TPM level) during the 6 months expsure.

(b)

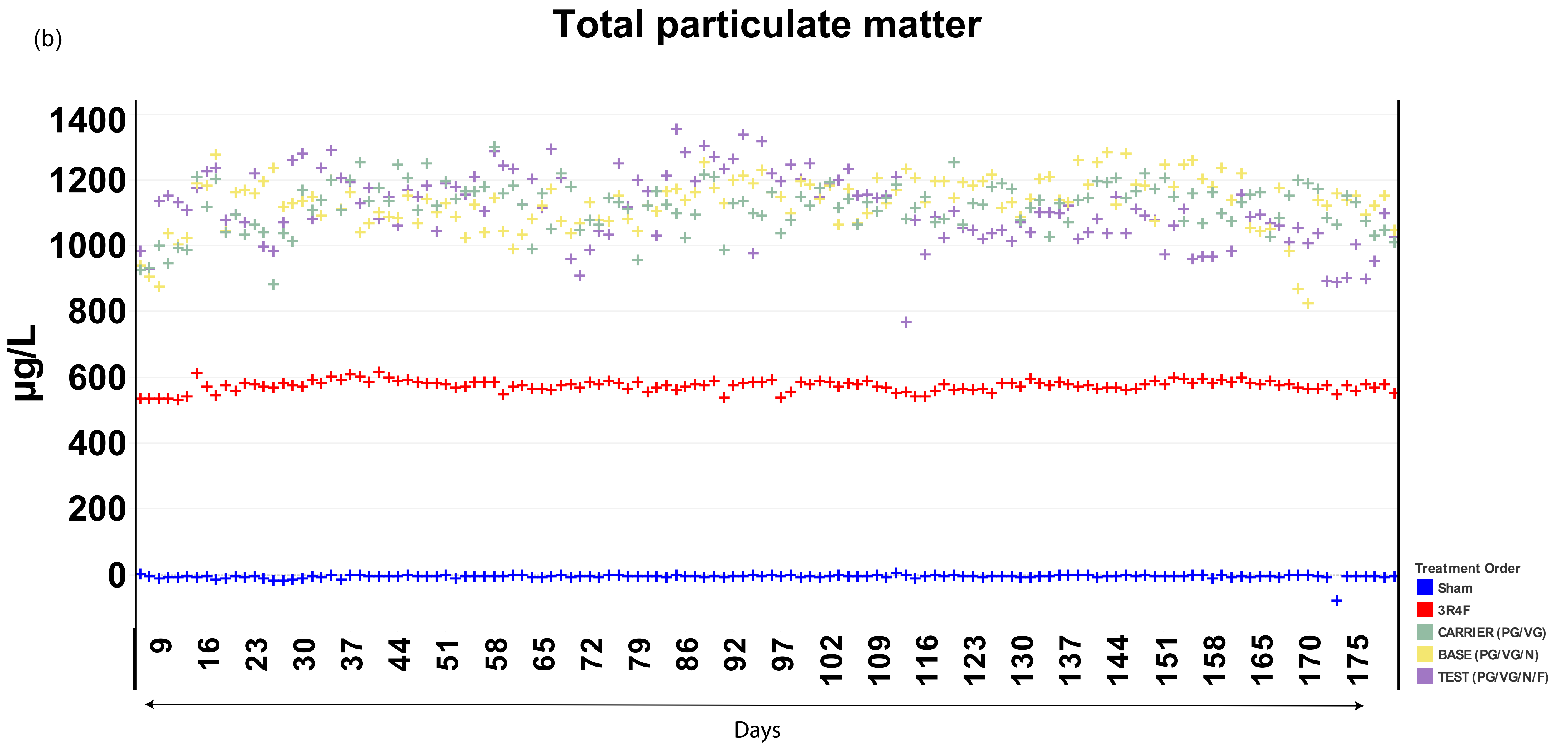

Online Resource 7: Longitudinal monitoring of nicotine and total particulate matter in the test atmosphere.
(a) Nicotine concentration. (b) Total particulate matter.

Online Resource 8

Region	Finding	Time	Sham 0	Sham 1	Sham 2	Sham 3	Sham 4	3R4F 0	3R4F 1	3R4F 2	3R4F 3	3R4F 4	CARRIER (PG/VG) 0	CARRIER (PG/VG) 1	CARRIER (PG/VG) 2	CARRIER (PG/VG) 3	CARRIER (PG/VG) 4	BASE (PG/VG/N) 0	BASE (PG/VG/N) 1	BASE (PG/VG/N) 2	BASE (PG/VG/N) 3	BASE (PG/VG/N) 4	TEST (PG/VG/N/F) 0	TEST (PG/VG/N/F) 1	TEST (PG/VG/N/F) 2	TEST (PG/VG/N/F) 3	TEST (PG/VG/N/F) 4
Left lung	Alveolar epithelium, hyperplasia	3M	12 / 12					5 / 12	7 / 12				12 / 12					12 / 12					12 / 12				
		6M	12 / 12					5 / 12	6 / 12	1 / 12			12 / 12					12 / 12					12 / 12				
	Alveolar interstitium/sub-pleural, lymphocytic cell aggregates	3M	12 / 12					12 / 12					12 / 12					11 / 12			1 / 12		12 / 12				
		6M	12 / 12					4 / 12	2 / 12	5 / 12	1 / 12		12 / 12					11 / 12	1 / 12				12 / 12				
	Alveolar lumen, hemorrhage	3M	12 / 12					12 / 12					12 / 12					12 / 12					12 / 12				
		6M	12 / 12					12 / 12					12 / 12					12 / 12					12 / 12				
	Alveolar lumen, lymphocytes/ plasma cells	3M	11 / 12	1 / 12				11 / 12	1 / 12				12 / 12					12 / 12					12 / 12				
		6M	12 / 12					7 / 12	5 / 12				12 / 12					11 / 12	1 / 12				11 / 12	1 / 12			
	Alveolar lumen, neutrophilic granulocytes	3M	12 / 12					1 / 12	11 / 12				11 / 12	1 / 12				12 / 12					12 / 12				
		6M	12 / 12					5 / 12	7 / 12				12 / 12					12 / 12					12 / 12				
	Alveolar lumen, pigmented macrophage nests	3M	12 / 12					3 / 12	8 / 12	1 / 12			12 / 12					12 / 12					12 / 12				
		6M	12 / 12					3 / 12	3 / 12	5 / 12	1 / 12		12 / 12					12 / 12					12 / 12				
	Alveolar lumen, transudate/exudate	3M	12 / 12					12 / 12					12 / 12					12 / 12					12 / 12				
		6M	12 / 12					12 / 12					12 / 12					12 / 12					12 / 12				
	Alveolar lumen, unpigmented macrophages	3M	10 / 12	2 / 12						1 / 12	11 / 12		9 / 12	3 / 12				8 / 12	4 / 12				9 / 12	3 / 12			
		6M	11 / 12	1 / 12					1 / 12	2 / 12	9 / 12		10 / 12	2 / 12				10 / 12	2 / 12				8 / 12	4 / 12			
	Alveolar lumen, yellow pigmented macrophages	3M	12 / 12					1 / 12	1 / 12	4 / 12	6 / 12		12 / 12					12 / 12					12 / 12				
		6M	12 / 12					1 / 12	1 / 12	1 / 12	4 / 12	5 / 12	12 / 12					12 / 12					12 / 12				
	Blood vessels, thrombus	3M	12 / 12					12 / 12					12 / 12					12 / 12					12 / 12				
		6M	12 / 12					12 / 12					12 / 12					12 / 12					12 / 12				
	Congestion of blood vessels	3M	12 / 12					12 / 12					12 / 12					12 / 12					12 / 12				
		6M	12 / 12					11 / 12	1 / 12				12 / 12					12 / 12					12 / 12				
	Emphysema	3M	10 / 12	2 / 12				2 / 12	8 / 12	2 / 12			10 / 12	2 / 12				12 / 12					10 / 12	2 / 12			
		6M	9 / 12	3 / 12				1 / 12	3 / 12	5 / 12	3 / 12		10 / 12	2 / 12				10 / 12	2 / 12				10 / 12	2 / 12			
	Main bronchus, goblet cell hyperplasia	3M	11 / 12		1 / 12			11 / 12	1 / 12				12 / 12					12 / 12					11 / 12	1 / 12			
		6M	10 / 12	1 / 12		1 / 12		12 / 12					12 / 12					11 / 12	1 / 12				11 / 12	1 / 12			
	Osseus metaplasia	3M	12 / 12					12 / 12					12 / 12					11 / 12	1 / 12				11 / 12	1 / 12			
		6M	12 / 12					12 / 12					12 / 12					11 / 12	1 / 12				12 / 12				
	Peri-vascular, mono-nuclear inflammatory cells	3M	10 / 12	1 / 12	1 / 12			7 / 12	4 / 12	1 / 12			9 / 12	2 / 12	1 / 12			10 / 12	1 / 12	1 / 12			9 / 12	2 / 12		1 / 12	
		6M	7 / 12	2 / 12	2 / 12		1 / 12	3 / 12	1 / 12	4 / 12	2 / 12	2 / 12	8 / 12	1 / 12	3 / 12			7 / 12	3 / 12		1 / 12	1 / 12	6 / 12	1 / 12	3 / 12		2 / 12
Nose level 1	Respiratory epithelium, degeneration	3M	10 / 12	1 / 12				12 / 12					9 / 12	3 / 12				11 / 12	1 / 12				10 / 12	2 / 12			
		6M	11 / 12	1 / 12				12 / 12					12 / 12					11 / 12	1 / 12				12 / 12				
	Respiratory epithelium, eosinophilic globules	3M	5 / 12	1 / 12	4 / 12	1 / 12		11 / 12	1 / 12				6 / 12	5 / 12	1 / 12			5 / 12	3 / 12	4 / 12			5 / 12	3 / 12	4 / 12		
		6M	10 / 12		2 / 12			10 / 12	2 / 12				9 / 12	2 / 12	1 / 12			8 / 12	4 / 12				9 / 12	1 / 12	2 / 12		
	Respiratory epithelium, hyperplasia	3M	6 / 12	5 / 12							5 / 12	7 / 12	6 / 12	5 / 12	1 / 12			7 / 12	4 / 12	1 / 12			7 / 12	5 / 12			
		6M	7 / 12	4 / 12		1 / 12					5 / 12	7 / 12	7 / 12	4 / 12	1 / 12			8 / 12	4 / 12				8 / 12	4 / 12			
	Respiratory epithelium, lamina propria, inflammatory cell infiltration	3M	11 / 12					12 / 12					12 / 12					12 / 12					12 / 12				
		6M	11 / 12	1 / 12				12 / 12					12 / 12					12 / 12					12 / 12				
	Respiratory epithelium, squamous epithelial metaplasia	3M	11 / 12							3 / 12	9 / 12		12 / 12					12 / 12					12 / 12				
		6M	12 / 12							2 / 12	10 / 12		12 / 12					12 / 12					12 / 12				
	Respiratory epithelium, sub-epithelial blood vessels, dilatation	3M	11 / 12					12 / 12					12 / 12					12 / 12					12 / 12				
		6M	11 / 12		1 / 12			12 / 12					12 / 12					12 / 12					12 / 12				
	Respiratory region, hyperplasia of goblet cells, septum	3M	10 / 12	1 / 12				12 / 12					11 / 12	1 / 12				10 / 12	2 / 12				11 / 12	1 / 12			
		6M	8 / 12	3 / 12		1 / 12		10 / 12	2 / 12				12 / 12					10 / 12	2 / 12				9 / 12	2 / 12	1 / 12		
	Respiratory region, lumen, amorphous eosinophilic material	3M	11 / 12					12 / 12					11 / 12	1 / 12				12 / 12					12 / 12				
		6M	12 / 12					12 / 12					12 / 12					12 / 12					12 / 12				
	Respiratory region, lumen, cell debris	3M	11 / 12					11 / 12	1 / 12				12 / 12					12 / 12					11 / 12		1 / 12		
		6M	11 / 12	1 / 12				12 / 12					12 / 12					12 / 12					12 / 12				
	Respiratory region, lumen, foreign material	3M	11 / 12					11 / 12	1 / 12				11 / 12	1 / 12				12 / 12					11 / 12		1 / 12		
		6M	12 / 12					12 / 12					12 / 12					12 / 12					12 / 12				
	Respiratory region, lumen, red blood cells	3M	11 / 12					12 / 12					12 / 12					12 / 12					12 / 12				
		6M	11 / 12	1 / 12				12 / 12					12 / 12					12 / 12					12 / 12				
Nose level 2	Olfactory epithelium, atrophy	3M	10 / 12	2 / 12				7 / 12	3 / 12	2 / 12			9 / 12	3 / 12				10 / 12	2 / 12				10 / 12	2 / 12			
		6M	12 / 12					8 / 12	2 / 12	2 / 12			12 / 12					12 / 12					12 / 12				
	Olfactory epithelium, lamina propria, loss of nerve bundles	3M	12 / 12					12 / 12					12 / 12					12 / 12					12 / 12				
		6M	12 / 12					11 / 12			1 / 12		12 / 12					12 / 12					12 / 12				
	Olfactory epithelium, lamina propria, lymphocytic cell aggregates	3M	12 / 12					12 / 12					12 / 12					12 / 12					12 / 12				
		6M	11 / 12			1 / 12		12 / 12					12 / 12					12 / 12					12 / 12				
	Respiratory epithelium, eosinophilic globules	3M	10 / 12		2 / 12			9 / 12	2 / 12	1 / 12			11 / 12	1 / 12				11 / 12	1 / 12				12 / 12				
		6M	12 / 12					12 / 12					11 / 12	1 / 12				12 / 12					12 / 12				
	Respiratory epithelium, hyperplasia	3M	12 / 12					9 / 12	3 / 12				12 / 12					12 / 12					12 / 12				
		6M	12 / 12					10 / 12	2 / 12				12 / 12					12 / 12					12 / 12				
	Respiratory epithelium, sub-mucosal gland, ectasia	3M	12 / 12					11 / 12	1 / 12				12 / 12					12 / 12					12 / 12				
		6M	11 / 12	1 / 12				12 / 12					12 / 12					12 / 12					12 / 12				
	Respiratory region, lumen, cell debris	3M	12 / 12					12 / 12					12 / 12					12 / 12					12 / 12				
		6M	11 / 12	1 / 12				12 / 12					12 / 12					11 / 12		1 / 12			12 / 12				
	Respiratory region, lumen, foreign material	3M	12 / 12					12 / 12					12 / 12					11 / 12		1 / 12			10 / 12	1 / 12	1 / 12		
		6M	12 / 12					12 / 12					12 / 12					10 / 12	1 / 12	1 / 12			11 / 12	1 / 12			
Nose level 3	Olfactory epithelium, atrophy	3M	12 / 12					12 / 12					12 / 12					12 / 12					12 / 12				
		6M	12 / 12					11 / 12	1 / 12				12 / 12					12 / 12					12 / 12				
	Olfactory epithelium, eosinophilic globules	3M	8 / 12	3 / 12	1 / 12			6 / 12	4 / 12	2 / 12			4 / 12		3 / 12	4 / 12	1 / 12	2 / 12	1 / 12	6 / 12	2 / 12	1 / 12	3 / 12	1 / 12	3 / 12	5 / 12	
		6M	9 / 12	3 / 12				4 / 12	6 / 12	1 / 12	1 / 12			1 / 12	2 / 12	8 / 12	1 / 12	1 / 12	3 / 12	5 / 12	3 / 12		1 / 12	3 / 12	1 / 12	7 / 12	
Nose level 4	Olfactory epithelium, eosinophilic globules	3M	12 / 12					11 / 12			1 / 12		5 / 12	1 / 12	3 / 12	2 / 12		3 / 12		4 / 12	4 / 12	1 / 12	3 / 12	2 / 12	3 / 12	3 / 12	1 / 12
		6M	11 / 12			1 / 12		10 / 12	1 / 12		1 / 12		3 / 12	4 / 12	5 / 12			4 / 12	4 / 12	4 / 12			3 / 12	6 / 12	3 / 12		
	Pharyngeal duct, epithelium, eosinophilic globules	3M	12 / 12					11 / 12		1 / 12			11 / 12					12 / 12					12 / 12				
		6M	12 / 12					12 / 12					12 / 12					12 / 12					12 / 12				
	Sub-mucosal gland, ectasis	3M	11 / 12	1 / 12				12 / 12					11 / 12					12 / 12					12 / 12				
		6M	12 / 12					12 / 12					12 / 12					12 / 12					12 / 12				

Histopathology respiratory

The number indicates the number of mice presenting the corresponding severity.

Incidence score
0 5

Online Resource 8: Histopathological incidences observed in the lungs and nasal tissues in response to 3R4F-CS or e-vapor aerosol exposure. (n=12)

Online Resource 9

Region	Finding	Measure	Time	Sham	3R4F	CARRIER (PG/VG)	BASE (PG/VG/N)	TEST (PG/VG/N/F)
Larynx - base of epiglottis	Epithelium, cornification	Score	3M	0.00 (± 0.00)	0.73 (± 1.35)	0.00 (± 0.00)	0.00 (± 0.00)	0.00 (± 0.00)
			6M	0.00 (± 0.00)	0.25 (± 0.62)	0.00 (± 0.00)	0.00 (± 0.00)	0.00 (± 0.00)
	Epithelium, degeneration/ ulceration	Score	3M	0.00 (± 0.00)	0.00 (± 0.00)	0.00 (± 0.00)	0.00 (± 0.00)	0.00 (± 0.00)
			6M	0.17 (± 0.39)	0.00 (± 0.00)	0.00 (± 0.00)	0.00 (± 0.00)	0.00 (± 0.00)
	Epithelium, hyperplasia	Score	3M	1.92 (± 0.90)	4.00 (± 0.00)bt+	2.33 (± 0.89)	2.00 (± 0.95)	2.33 (± 0.78)
			6M	1.75 (± 1.14)	4.00 (± 0.43)bt+	2.42 (± 0.79)	1.83 (± 0.83)	2.25 (± 1.06)
	Epithelium, squamous epithelial metaplasia	Score	3M	0.25 (± 0.45)	3.27 (± 0.65)bt+	0.92 (± 0.79)+	0.75 (± 0.87)	0.92 (± 0.90)
			6M	0.50 (± 0.80)	3.33 (± 0.89)bt+	0.92 (± 0.90)	0.58 (± 0.67)	1.42 (± 1.08)b+
	Lumen, amorphous eosinophilic material	Score	3M	0.00 (± 0.00)	0.00 (± 0.00)	0.00 (± 0.00)	0.00 (± 0.00)	0.00 (± 0.00)
			6M	0.00 (± 0.00)	0.00 (± 0.00)	0.00 (± 0.00)	0.00 (± 0.00)	0.08 (± 0.29)
	Lumen, red blood cells	Score	3M	0.00 (± 0.00)	0.09 (± 0.30)	0.00 (± 0.00)	0.00 (± 0.00)	0.00 (± 0.00)
			6M	0.00 (± 0.00)	0.00 (± 0.00)	0.00 (± 0.00)	0.00 (± 0.00)	0.08 (± 0.29)
	Sub-mucosal gland/duct, ectasia/congestion	Score	3M	0.00 (± 0.00)	0.00 (± 0.00)	0.00 (± 0.00)	0.00 (± 0.00)	0.00 (± 0.00)
			6M	0.00 (± 0.00)	0.00 (± 0.00)	0.17 (± 0.58)	0.00 (± 0.00)	0.00 (± 0.00)
Larynx - floor	Epithelium, cornification	Score	3M	0.00 (± 0.00)	0.00 (± 0.00)	0.00 (± 0.00)	0.00 (± 0.00)	0.00 (± 0.00)
			6M	0.00 (± 0.00)	0.00 (± 0.00)	0.00 (± 0.00)	0.00 (± 0.00)	0.00 (± 0.00)
	Epithelium, degeneration	Score	3M	0.00 (± 0.00)	0.00 (± 0.00)	0.00 (± 0.00)	0.00 (± 0.00)	0.00 (± 0.00)
			6M	0.00 (± 0.00)	0.17 (± 0.58)	0.00 (± 0.00)	0.00 (± 0.00)	0.08 (± 0.29)
	Epithelium, hyperplasia	Score	3M	1.58 (± 1.08)	3.33 (± 0.49)bt+	1.91 (± 0.83)	2.00 (± 0.87)	1.92 (± 0.90)
			6M	1.64 (± 1.21)	3.50 (± 0.52)bt+	2.25 (± 1.14)	1.27 (± 1.19)	1.67 (± 1.07)
	Epithelium, squamous epithelial metaplasia	Score	3M	0.25 (± 0.45)	1.42 (± 0.90)bt+	0.18 (± 0.40)	0.11 (± 0.33)	0.50 (± 0.52)
			6M	0.27 (± 0.65)	1.42 (± 0.79)bt+	0.17 (± 0.39)	0.18 (± 0.60)	0.00 (± 0.00)
Larynx - ventral depression	Epithelium, lamina propria, inflammatory cell infiltration	Score	3M	0.00 (± 0.00)	0.00 (± 0.00)	0.00 (± 0.00)	0.00 (± 0.00)	0.00 (± 0.00)
			6M	0.00 (± 0.00)	0.00 (± 0.00)	0.00 (± 0.00)	0.09 (± 0.30)	0.00 (± 0.00)
	Epithelium, squamous epithelial metaplasia	Score	3M	0.00 (± 0.00)	0.00 (± 0.00)	0.00 (± 0.00)	0.00 (± 0.00)	0.00 (± 0.00)
			6M	0.00 (± 0.00)	0.00 (± 0.00)	0.00 (± 0.00)	0.00 (± 0.00)	0.00 (± 0.00)
	Epithelium, ulceration	Score	3M	0.00 (± 0.00)	0.00 (± 0.00)	0.00 (± 0.00)	0.00 (± 0.00)	0.00 (± 0.00)
			6M	0.00 (± 0.00)	0.00 (± 0.00)	0.00 (± 0.00)	0.00 (± 0.00)	0.00 (± 0.00)
	Lumen, foreign material	Score	3M	0.00 (± 0.00)	0.00 (± 0.00)	0.00 (± 0.00)	0.22 (± 0.67)	0.00 (± 0.00)
			6M	0.00 (± 0.00)	0.00 (± 0.00)	0.00 (± 0.00)	0.27 (± 0.90)	0.00 (± 0.00)
Larynx - vocal cords	Epithelium, cornification	Score	3M	0.00 (± 0.00)	0.00 (± 0.00)	0.00 (± 0.00)	0.00 (± 0.00)	0.09 (± 0.30)
			6M	0.00 (± 0.00)	0.18 (± 0.40)	0.00 (± 0.00)	0.00 (± 0.00)	0.00 (± 0.00)
	Epithelium, hyperplasia	Score	3M	2.09 (± 0.70)	3.18 (± 0.75)bt+	2.20 (± 1.03)	1.75 (± 0.89)	2.18 (± 0.87)
			6M	1.55 (± 1.37)	3.45 (± 0.69)bt+	2.00 (± 0.85)	1.18 (± 0.87)c	1.09 (± 1.14)
Trachea - transverse section	Epithelium, hyperplasia	Score	3M	0.00 (± 0.00)	0.33 (± 0.65)	0.17 (± 0.39)	0.18 (± 0.40)	0.17 (± 0.39)
			6M	0.00 (± 0.00)	0.00 (± 0.00)	0.08 (± 0.29)	0.00 (± 0.00)	0.08 (± 0.29)
	Epithelium, squamous epithelial metaplasia	Score	3M	0.00 (± 0.00)	0.00 (± 0.00)	0.00 (± 0.00)	0.09 (± 0.30)	0.00 (± 0.00)
			6M	0.00 (± 0.00)	0.00 (± 0.00)	0.08 (± 0.29)	0.00 (± 0.00)	0.00 (± 0.00)
	Lumen, cell debris	Score	3M	0.00 (± 0.00)	0.00 (± 0.00)	0.00 (± 0.00)	0.00 (± 0.00)	0.00 (± 0.00)
			6M	0.08 (± 0.29)	0.00 (± 0.00)	0.00 (± 0.00)	0.00 (± 0.00)	0.00 (± 0.00)
	Subepithelial gland, ectasia	Score	3M	0.00 (± 0.00)	0.00 (± 0.00)	0.00 (± 0.00)	0.00 (± 0.00)	0.00 (± 0.00)
			6M	0.00 (± 0.00)	0.00 (± 0.00)	0.00 (± 0.00)	0.08 (± 0.29)	0.00 (± 0.00)

+ p < 0.05 significant versus Sham
c p < 0.05 significant versus CARRIER
t p < 0.05 significant versus TEST
b p < 0.05 significant versus BASE
(M :Months)

Online Resource 9: Histopathological evaluation of changes in the larynx and trachea in response to 3R4F cigarette smoke (CS) or e-vapor aerosol exposure.
Data are presented as mean ± SD; n = 11–12. "+" denotes p < 0.05 versus the sham group; "t" denotes p < 0.05 versus the test group; "b" denotes p < 0.05 versus the base group. The histopathological score definitions were: (1) minimal; (2) mild; (3) moderate; (4) marked; and (5) severe.

Online Resource 10

Lung absolute and relative weights to brain and body weights.

			Sham	3R4F	CARRIER (PG/VG)	BASE (PG/VG/N)	TEST (PG/VG/N/F)
**Absolute weight after exangination**	g	**3M**	0.142 *(± 0.01)*	0.177 *(± 0.03)bt+*	0.142 *(± 0.01)*	0.142 *(± 0.01)*	0.142 *(± 0.01)*
		**6M**	0.146 *(± 0.01)*	0.179 *(± 0.03)bt+*	0.145 *(± 0.01)*	0.143 *(± 0.01)*	0.144 *(± 0.01)*
**Weight relative to body weight**	%	**3M**	0.569 *(± 0.03)*	0.763 *(± 0.14)bt+*	0.588 *(± 0.07)*	0.558 *(± 0.04)*	0.568 *(± 0.04)*
		**6M**	0.574 *(± 0.05)*	0.731 *(± 0.11)bt+*	0.555 *(± 0.04)*	0.523 *(± 0.04)+*	0.536 *(± 0.05)*
**Weight relative to brain weight**	%	**3M**	29.830 *(± 1.84)*	38.382 *(± 5.79)bt+*	30.409 *(± 2.35)*	29.781 *(± 2.24)*	30.188 *(± 1.62)*
		**6M**	31.230 *(± 3.90)*	37.337 *(± 5.78)bt+*	30.403 *(± 2.51)*	30.190 *(± 1.83)*	29.736 *(± 1.59)*

+ $p < 0.05$ significant versus Sham
c $p < 0.05$ significant versus CARRIER
t $p < 0.05$ significant versus TEST
b $p < 0.05$ significant versus BASE
(M :Months)

Online Resource 11

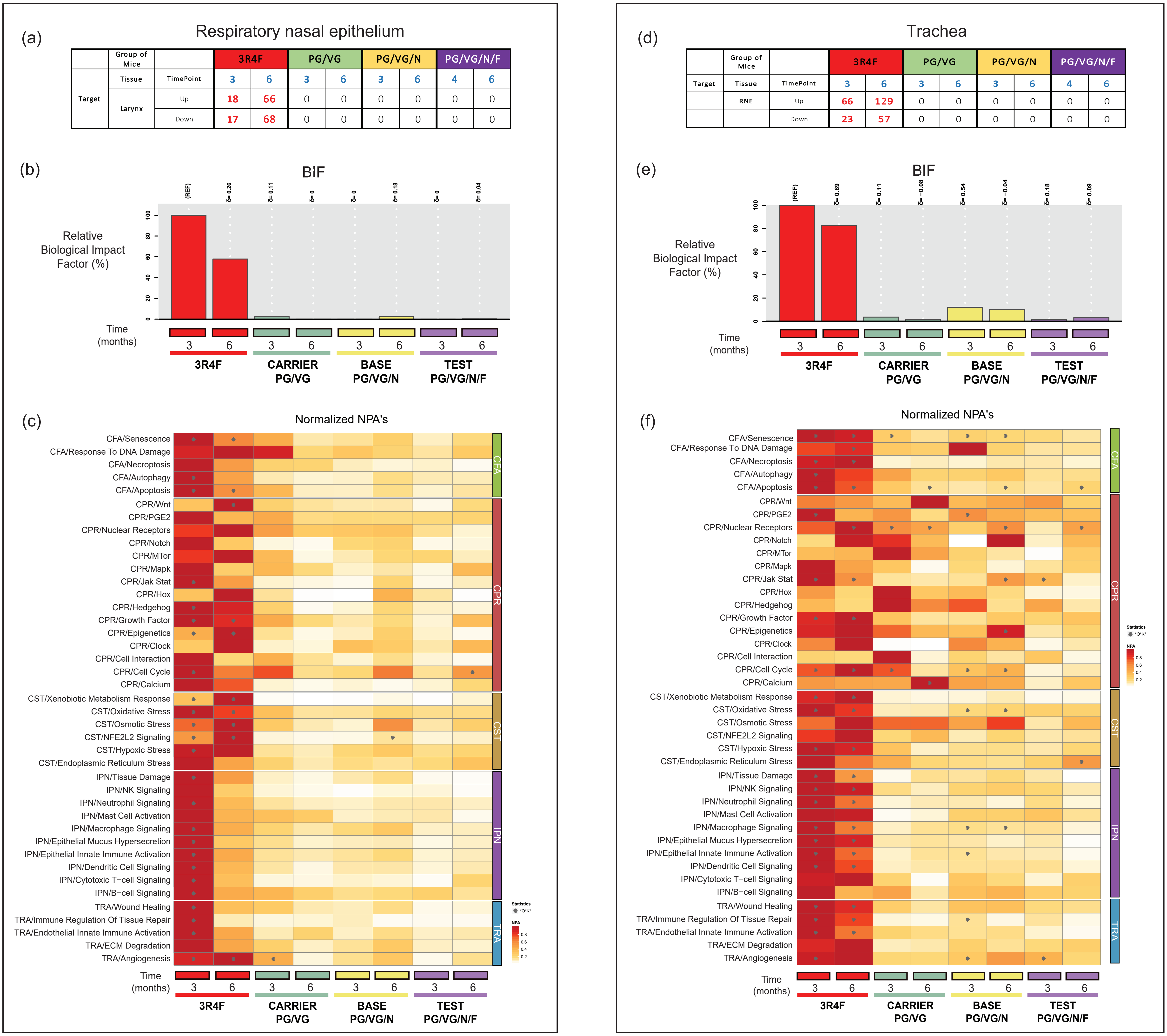

(a) Respiratory nasal epithelium

	Group of Mice		3R4F		PG/VG		PG/VG/N		PG/VG/N/F	
Target	Tissue	TimePoint	3	6	3	6	3	6	4	6
	Larynx	Up	18	66	0	0	0	0	0	0
		Down	17	68	0	0	0	0	0	0

(d) Trachea

	Group of Mice		3R4F		PG/VG		PG/VG/N		PG/VG/N/F	
Target	Tissue	TimePoint	3	6	3	6	3	6	4	6
	RNE	Up	66	129	0	0	0	0	0	0
		Down	23	57	0	0	0	0	0	0

Online Resource 11: Systems toxicology analysis of differentially expressed genes in the upper respiratory tract (nose and trachea).
(a) Number of differentially expressed genes; (b) Relative biological impact factor (RBIF) for treatment versus sham. The percentages show the RBIF, which is derived from the cumulated network perturbations caused by the treatment relative to the reference, defined as the treatment comparison showing the highest perturbation (i.e., at the 6-month time point). (c) Heatmap of network perturbation amplitude (NPA) scores summarizing subnetwork NPAs relative to the maximum NPA in each subnetwork. Stars indicate significant perturbations: A network is considered perturbed if, in addition to the significance of the NPA score with respect to the experimental variation, the two companion statistics (O and K), derived to inform on the specificity of the NPA score with respect to the biology described in the network, are significant. *O and K statistic p values <0.05 and significant with respect to the experimental variation; n = 9–10.

Online Resource 12

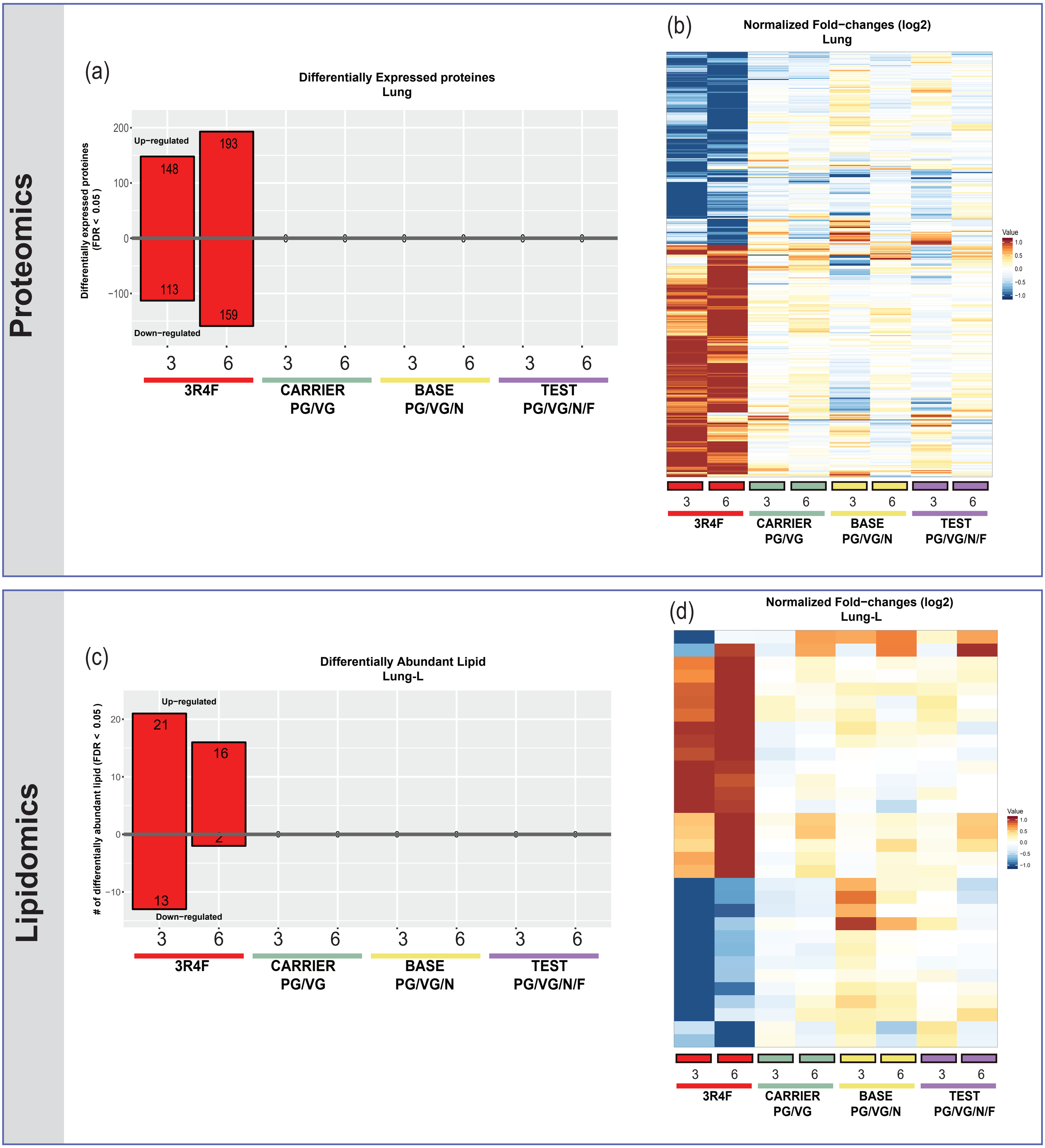

Online Resource 12: Systems toxicology analysis of lipids and proteins in the lungs.
(a) Number of differentially abundant proteins in the lungs compared with the numbers in the respective sham group of the same exposure month (false discovery rate [FDR]-adjusted p < 0.05). (b) Heatmap of the protein abundance fold changes. (c) Number of differentially abundant lipids in the lungs compared with the numbers in the respective sham group of the same exposure month (FDR-adjusted p < 0.05). (d) Heatmap of lipid abundance. Fold changes (log2, normalized to the maximum observed absolute value) that are significant in at least one comparison in lung tissues are shown. Red indicates genes, lipids, and proteins that are upregulated/increased, and blue indicates those that are significantly downregulated/decreased relative to the levels in the sham group at each respective time point.

Online Resource 13

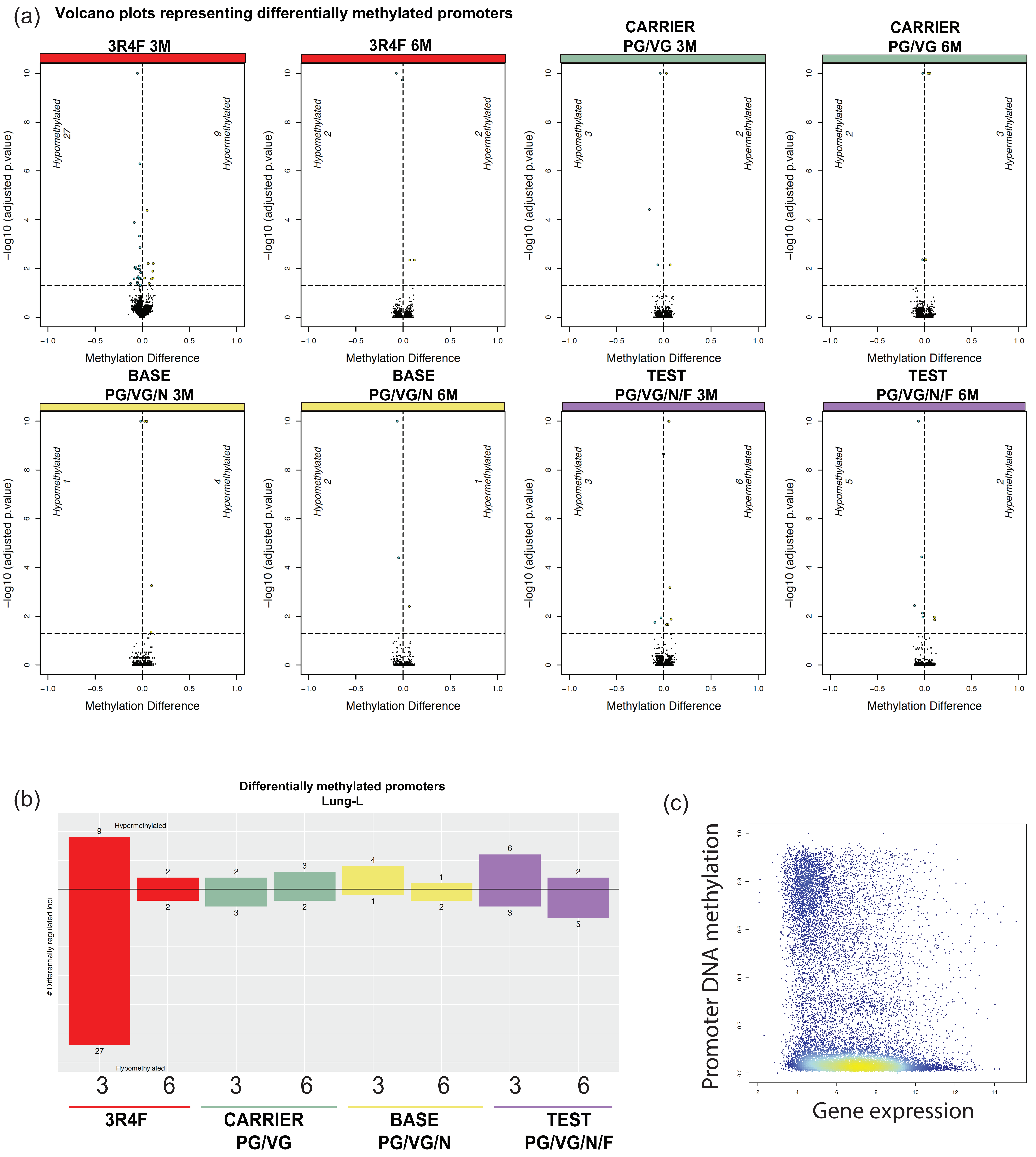

Online Resource 13: Systems toxicology assessment of DNA methylation changes at promoters in the lungs.
(a) Volcano plots representing differentially methylated promoters as the amplitude and significance of methylation changes at promoters between the indicated treatment and the respective control samples. Methylation difference (exposure minus control) is plotted on the x-axis, and the statistical significance—calculated as −log10 (false discovery rate [FDR]-adjusted p value)—is plotted on the y-axis. Yellow and blue dots indicate hyper- and hypomethylated loci, respectively, relative to the control samples. (b) Bar plot representing the number of differentially methylated promoters on the basis of the FDR cutoff (0.05). Sham samples were used as controls. (c) Scatterplot indicating the general profile of the correlation between DNA methylation at promoters and expression of the corresponding genes.

Online Resource 14

(a) **Differentially methylated candidate enhancers**

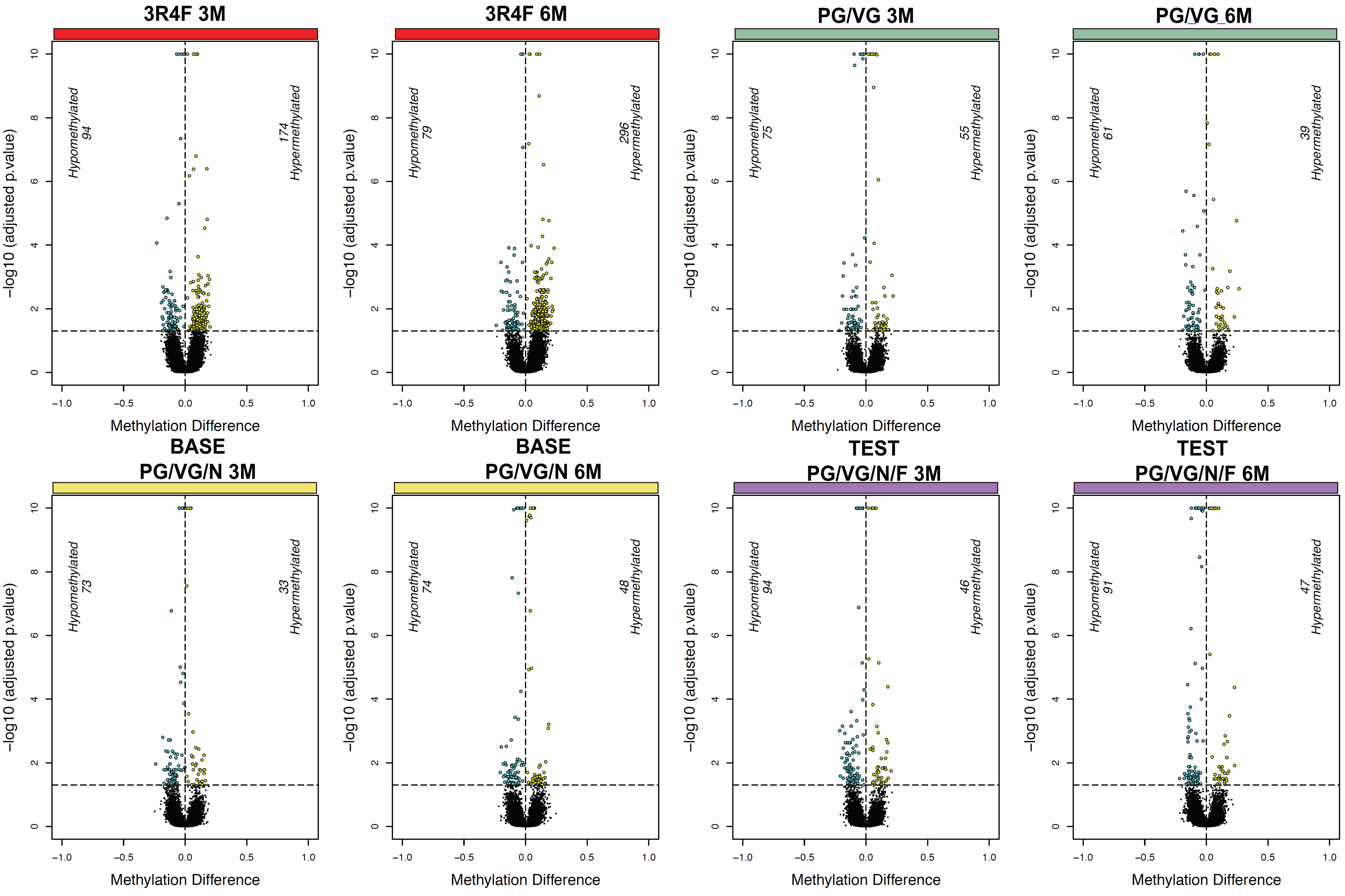

(b)

Differentially methylated candidate enhancers
Lung-L

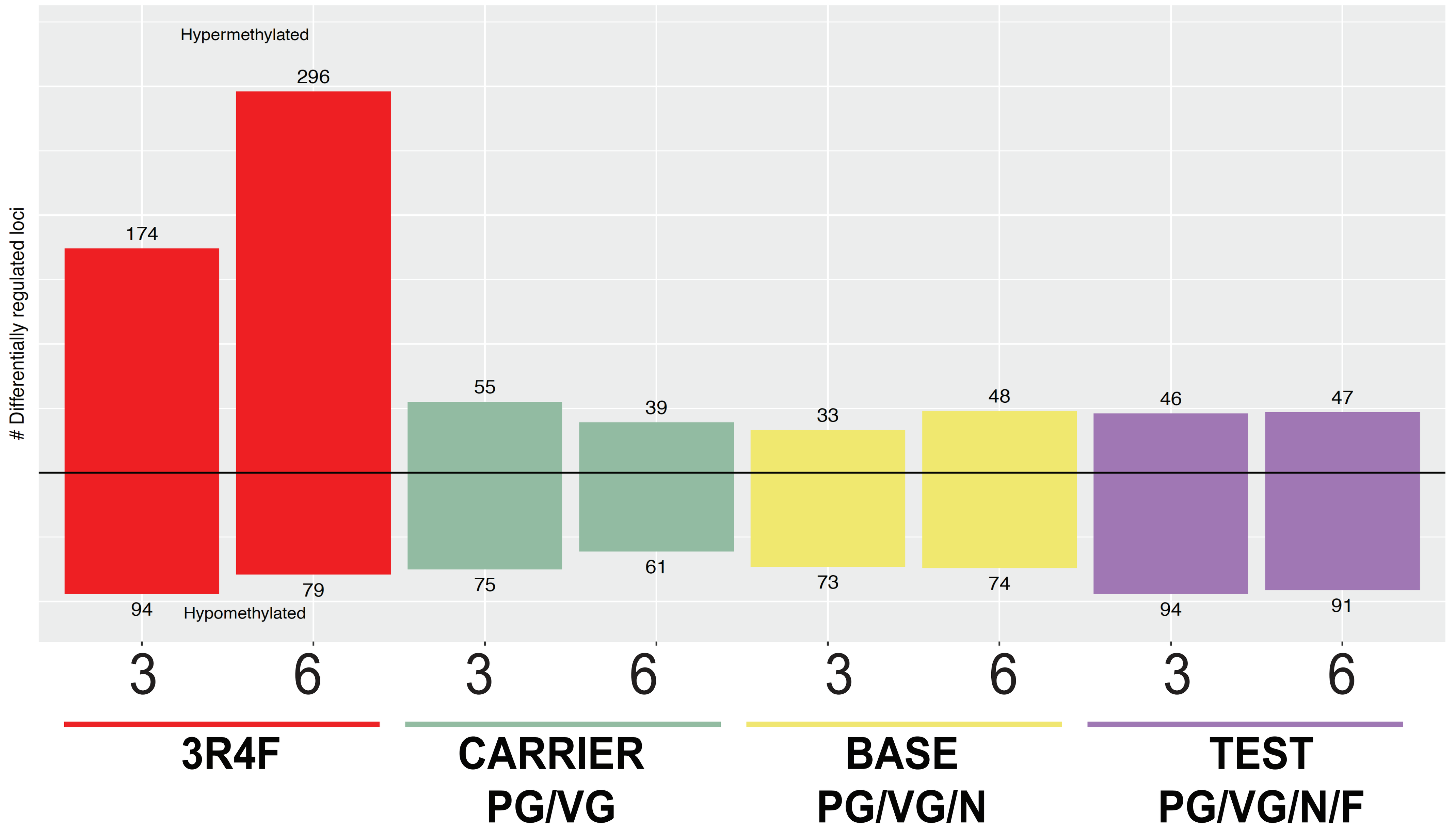

Online Resource 14: Systems toxicology assessment of DNA methylation changes at candidate enhancers in the lungs. (a) Volcano plots representing differentially methylated candidate enhancers as the amplitude and significance of methylation changes at candidate enhancers between the indicated treatment and the respective controls. Methylation difference (exposure minus control) is plotted on the x-axis, and the statistical significance—calculated as −log10 (false discovery rate [FDR]-adjusted p value)—is plotted on the y-axis. Yellow and blue dots indicate hyper- and hypomethylated loci, respectively, relative to the control samples. (b) Bar plot representing the number of differentially methylated candidate enhancers on the basis of the FDR cutoff (0.05). Sham samples were used as controls.
